# Supplementary figures and images for: Core motifs predict dynamic attractors in combinatorial threshold-linear networks
Source: PLoS One. 2022 Mar 4;17(3):e0264456. doi: 10.1371/journal.pone.0264456 (PMC8896682; doi:10.1371/journal.pone.0264456)

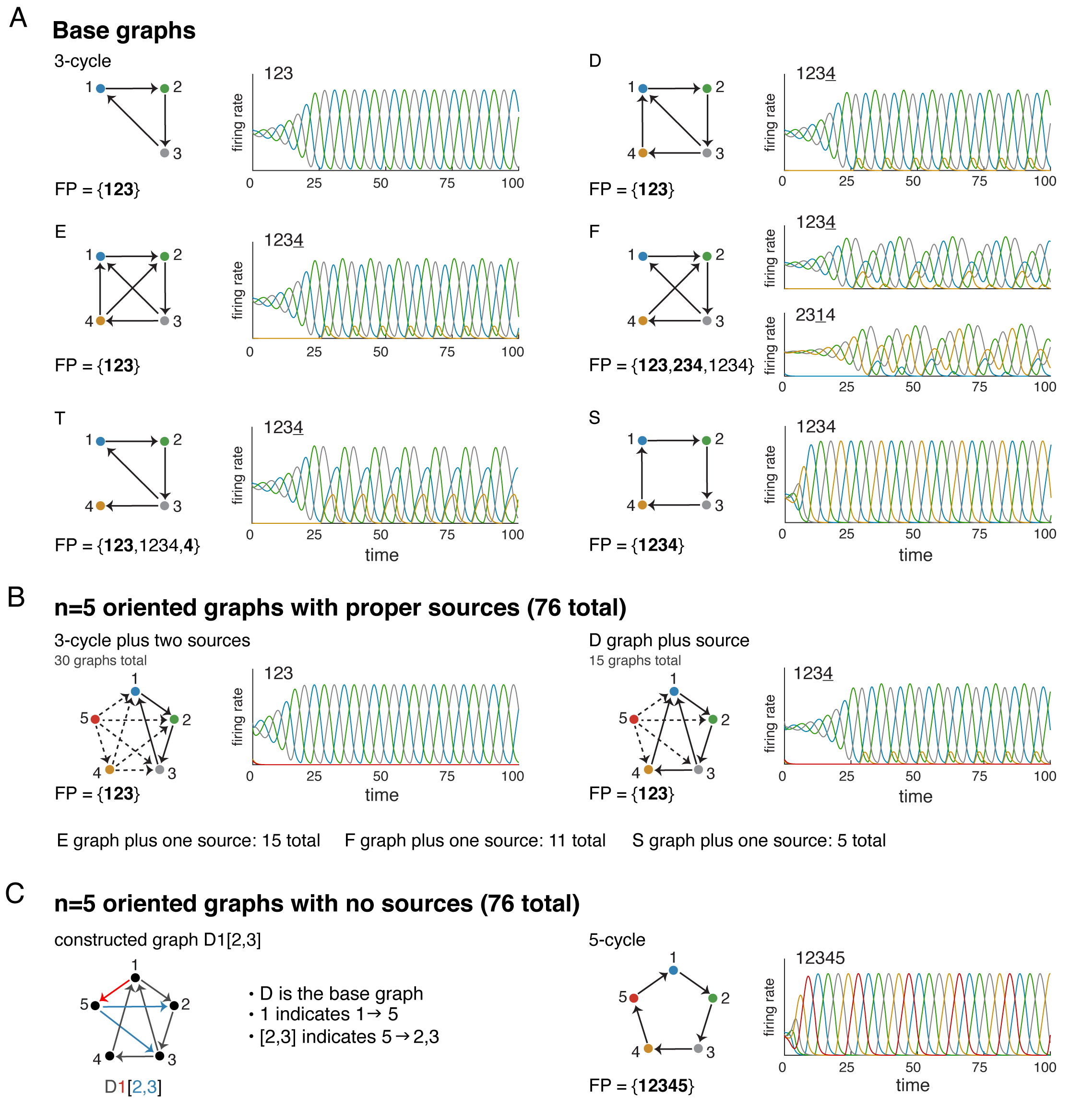

Supplement: S1 Fig — (A) Base graphs used to construct n = 5 graphs, and their corresponding attractors. Each attractor has a sequence, indicating the (periodic) order in which the neurons achieve their peak firing rates. (B) The oriented graphs with sources can be constructed by adding proper sources to each of the base graphs. This yields 30 graphs from the 3-cycle base (left), 15 graphs from the D graph base (right), and an additional 15, 11, and 5 graphs from the E, F and S graph bases. (C) All oriented graphs with no sources or sinks can be constructed from one of the D, E, F, T, and S base graphs. (Left) For example, D1[2, 3] is the graph constructed from the D graph with added edges 1 → 5 and 5 → 2, 3. (Right) The only oriented n = 5 graph with no sources or sinks that cannot be constructed in this way is the 5-cycle. (Same as Fig 6 in the main text). (TIF) [file pone.0264456.s001.tif]

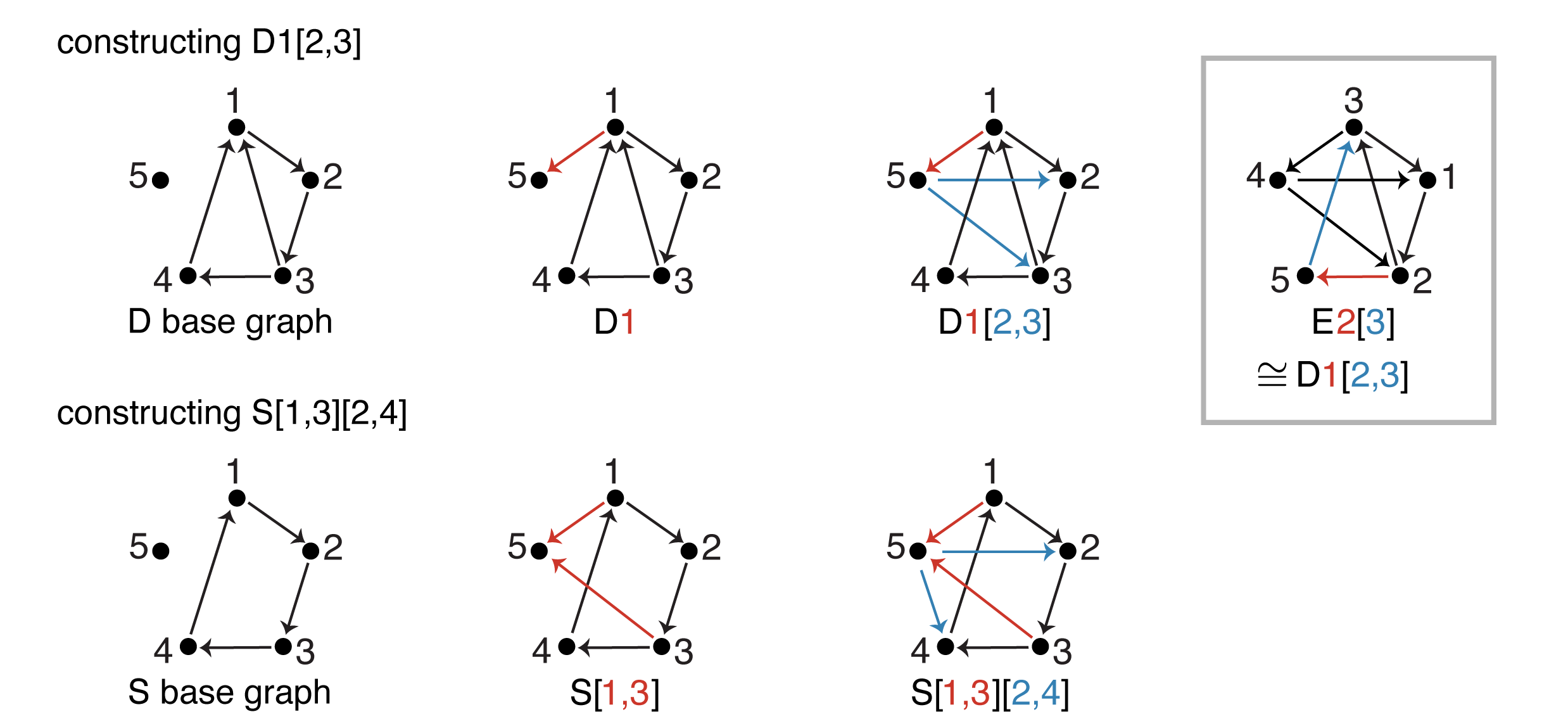

Supplement: S2 Fig — (Top) Starting with a D base, the graph D1[2, 3] is constructed by adding a node 5 together with incoming edge 1 → 5 (red) and outgoing edges 5 → 2 and 5 → 3 (blue). An isomorphic graph, E2[3], can be constructed from an E base. (Bottom) The graph S[1,3][2,4] has two incoming edges to node 5, given in the first set of brackets. This graph cannot be constructed from any base with only one edge into node 5. (TIF) [file pone.0264456.s002.tif]

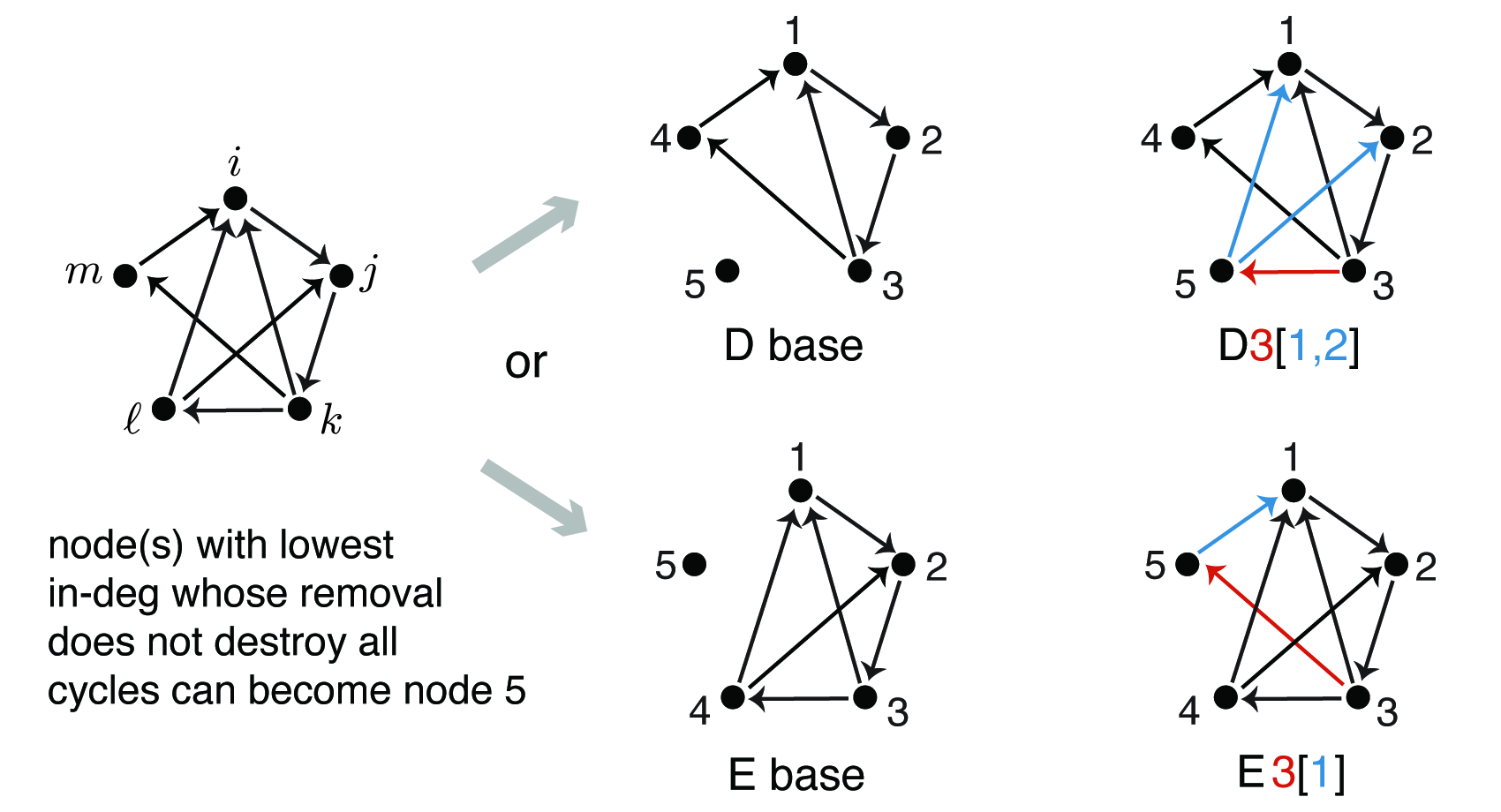

Supplement: S3 Fig — The nodes with lowest in-degree are k, ℓ, and m. However, removing k results in a graph with no cycles that cannot match one of our base graphs. Removing ℓ (top) uncovers a D graph base, while removing m (bottom) results in an E base. The original graph can thus be labeled as D3[1, 2] or E3[1]. (TIF) [file pone.0264456.s003.tif]

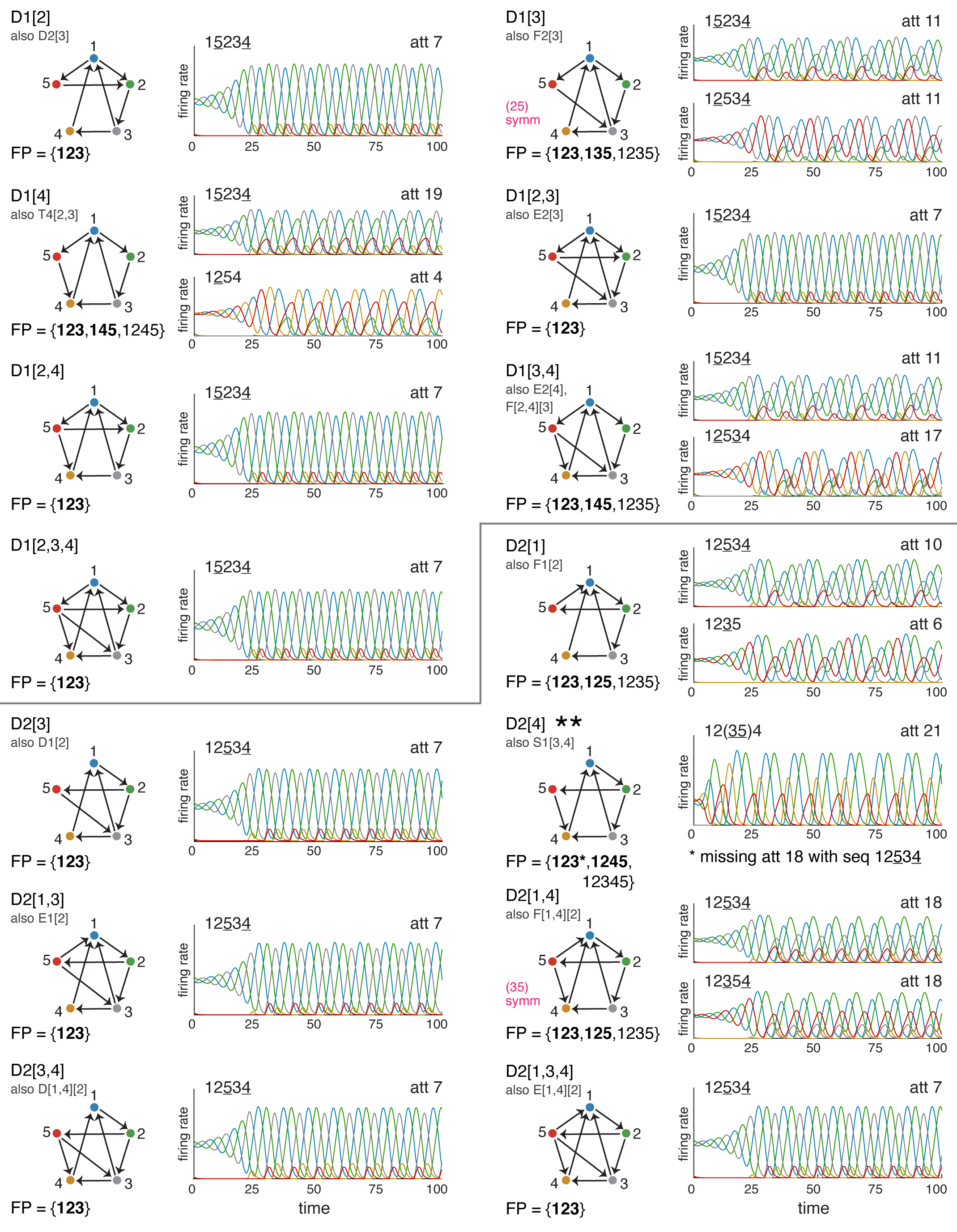

Supplement: S4 Fig — (TIF) [file pone.0264456.s004.tif]

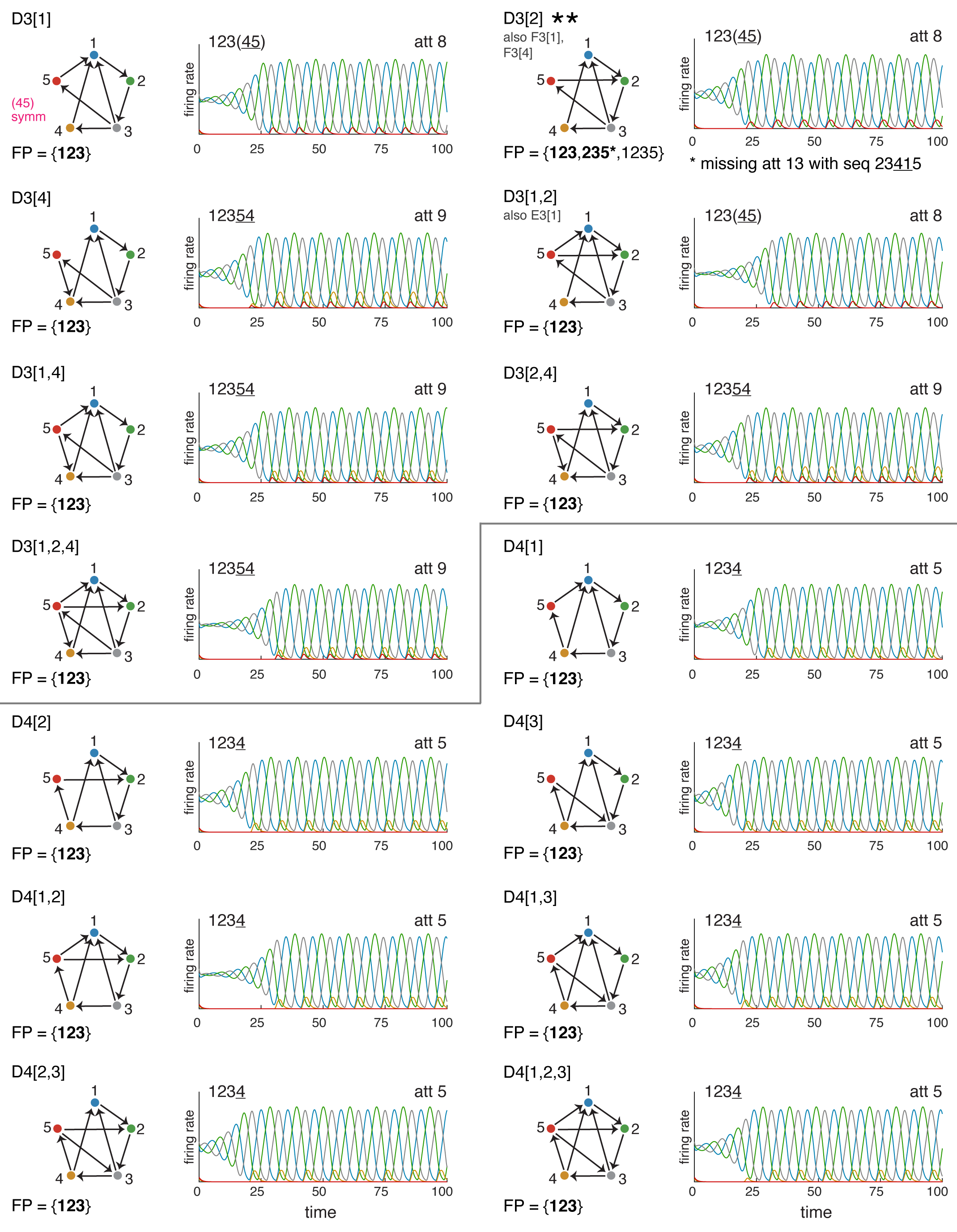

Supplement: S5 Fig — (TIF) [file pone.0264456.s005.tif]

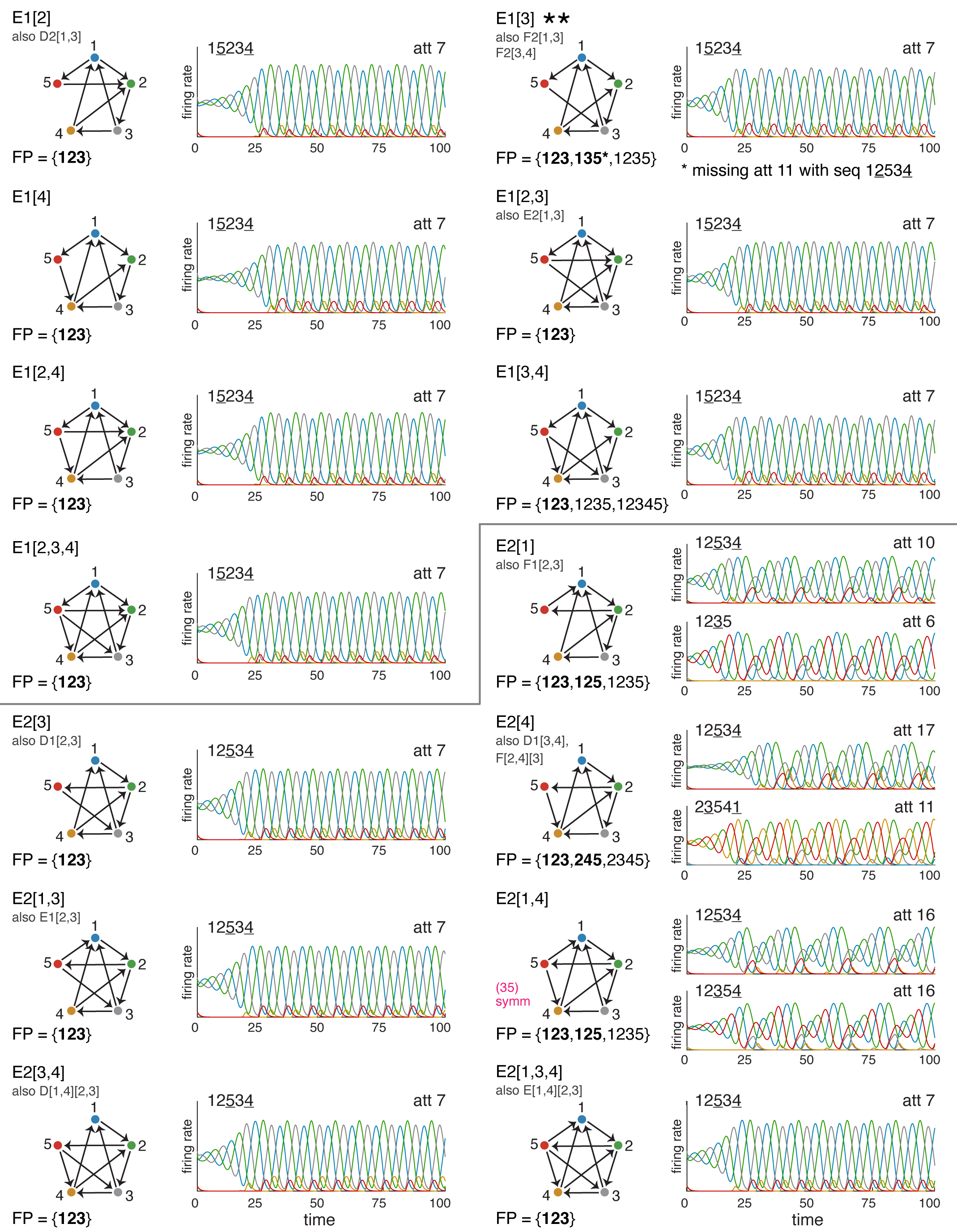

Supplement: S6 Fig — (TIF) [file pone.0264456.s006.tif]

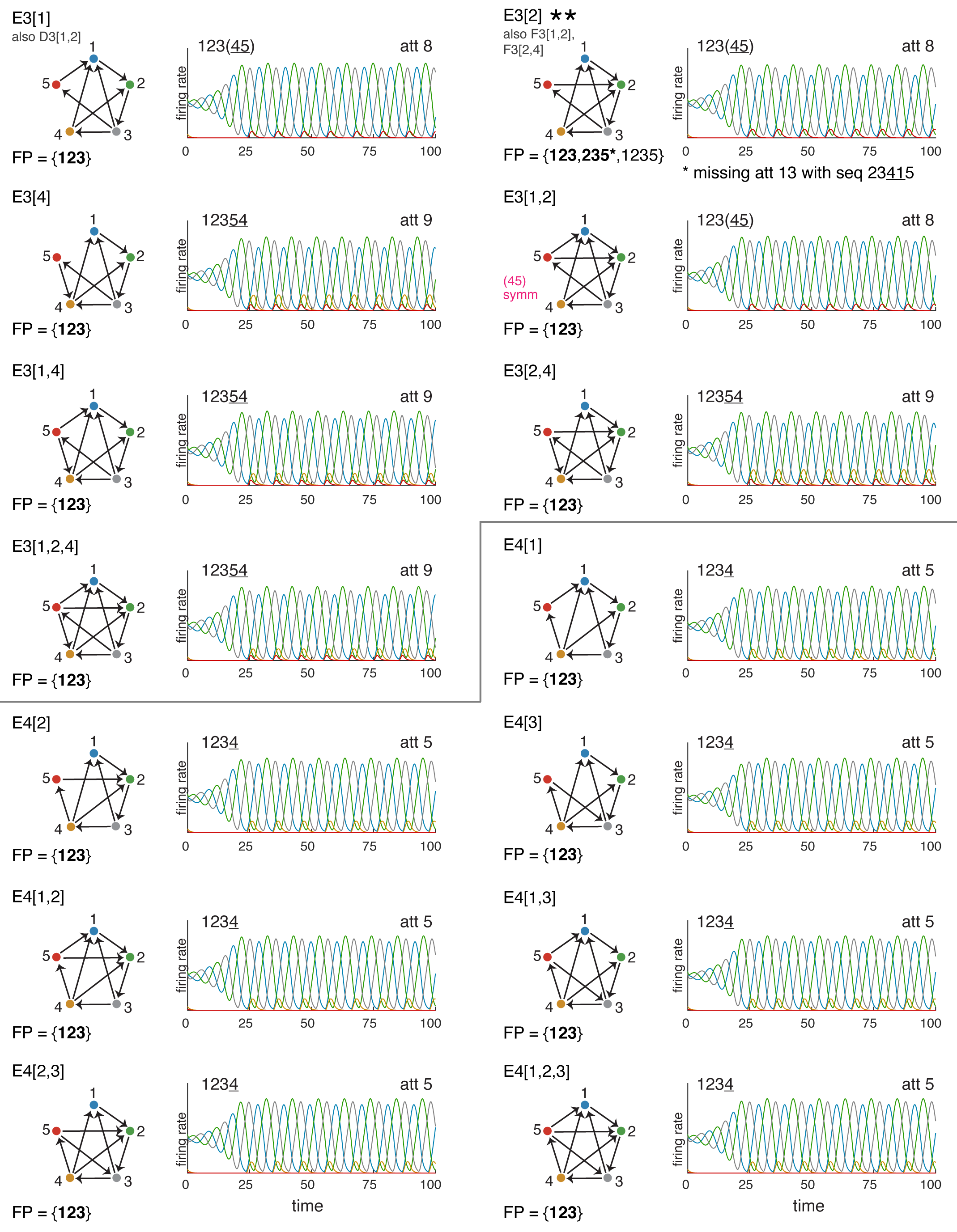

Supplement: S7 Fig — (TIF) [file pone.0264456.s007.tif]

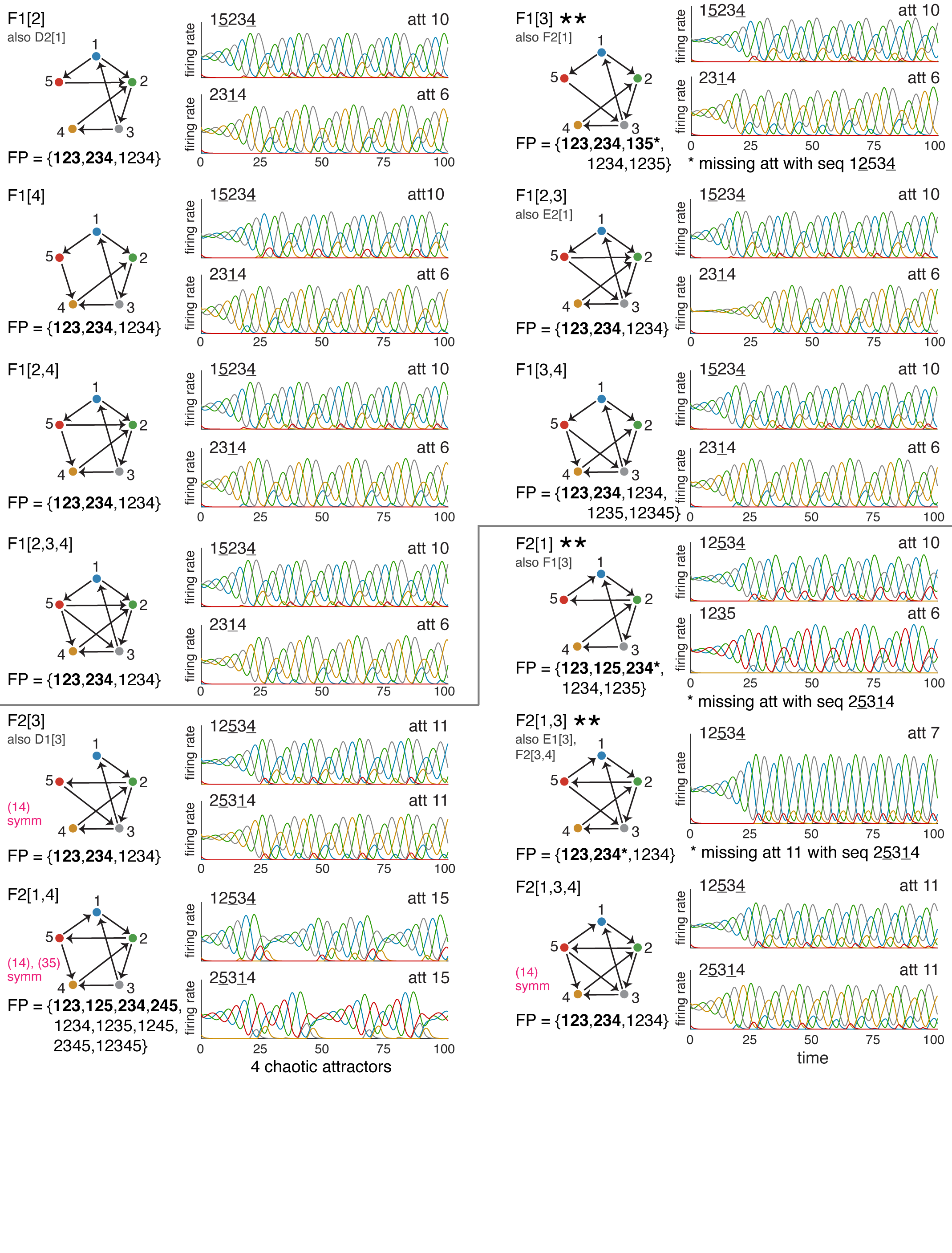

Supplement: S8 Fig — (TIF) [file pone.0264456.s008.tif]

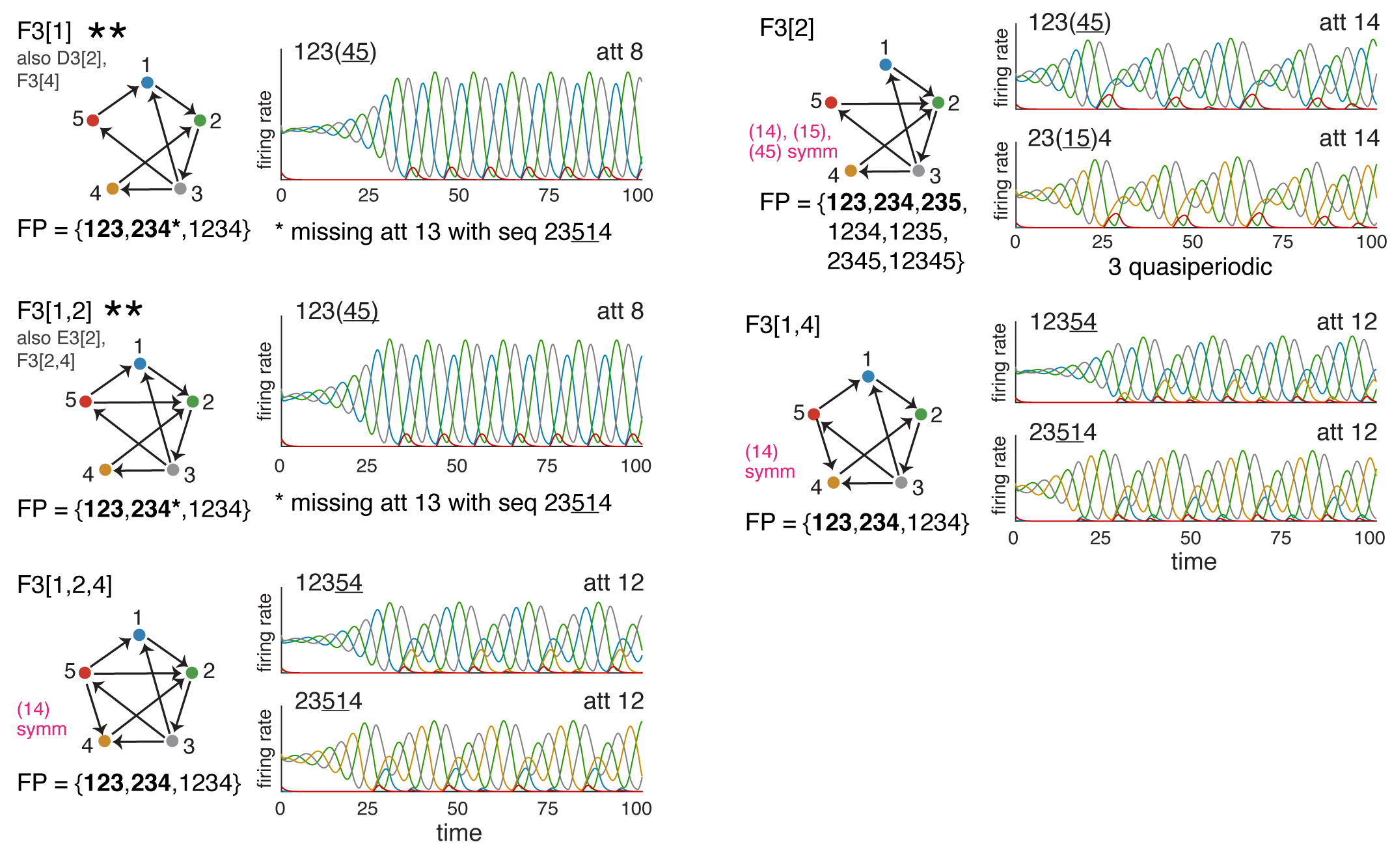

Supplement: S9 Fig — (TIF) [file pone.0264456.s009.tif]

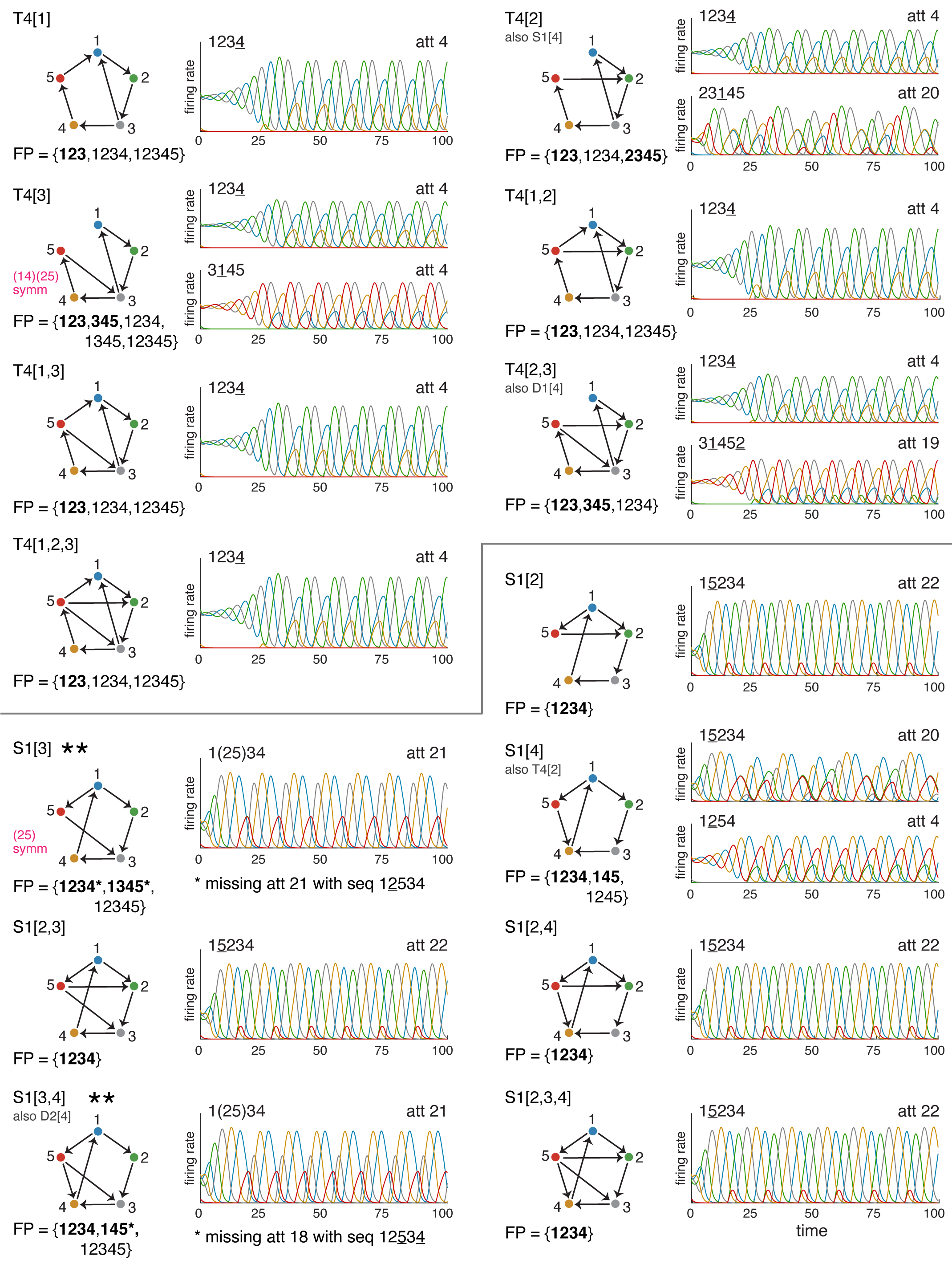

Supplement: S10 Fig — (TIF) [file pone.0264456.s010.tif]

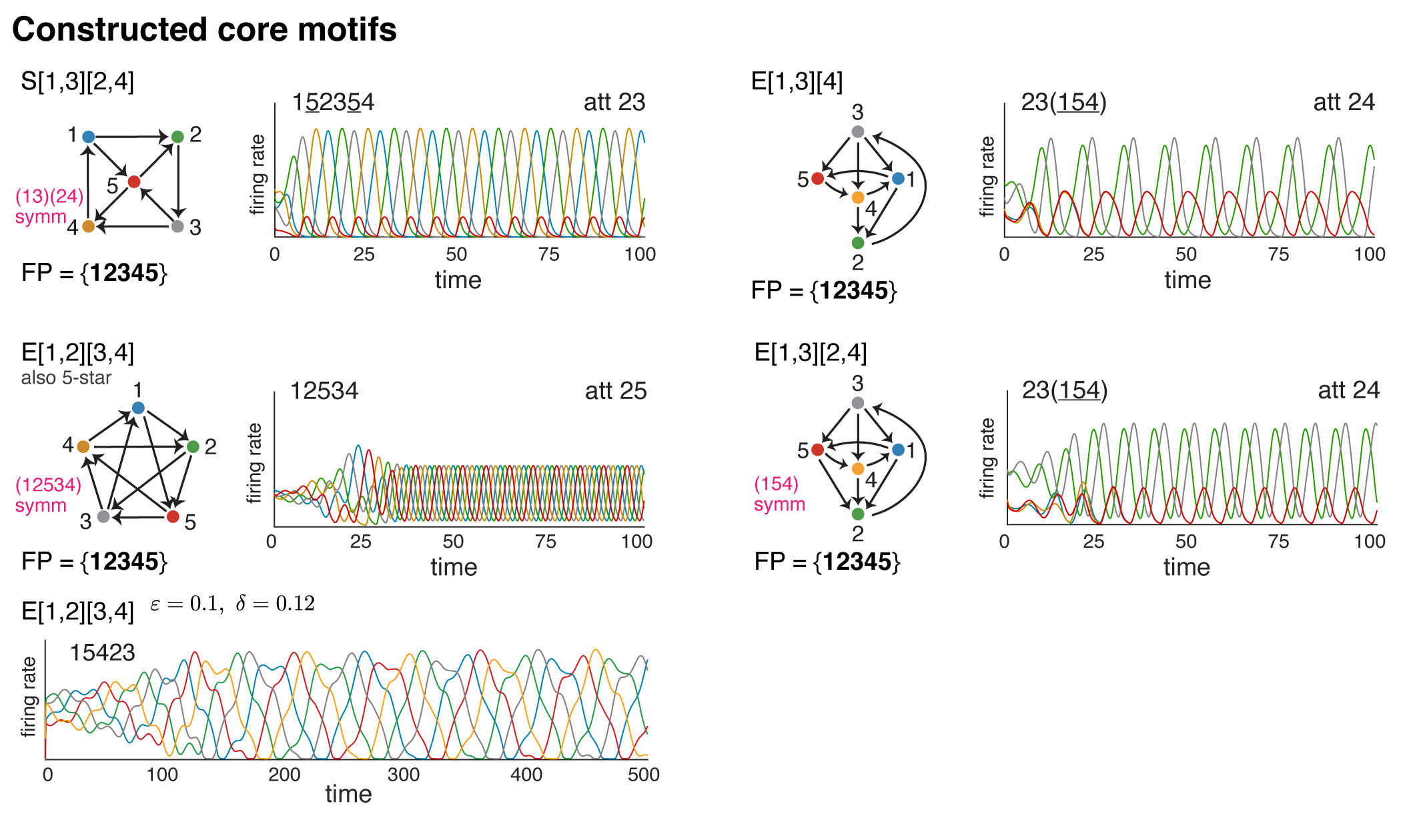

Supplement: S11 Fig — (TIF) [file pone.0264456.s011.tif]

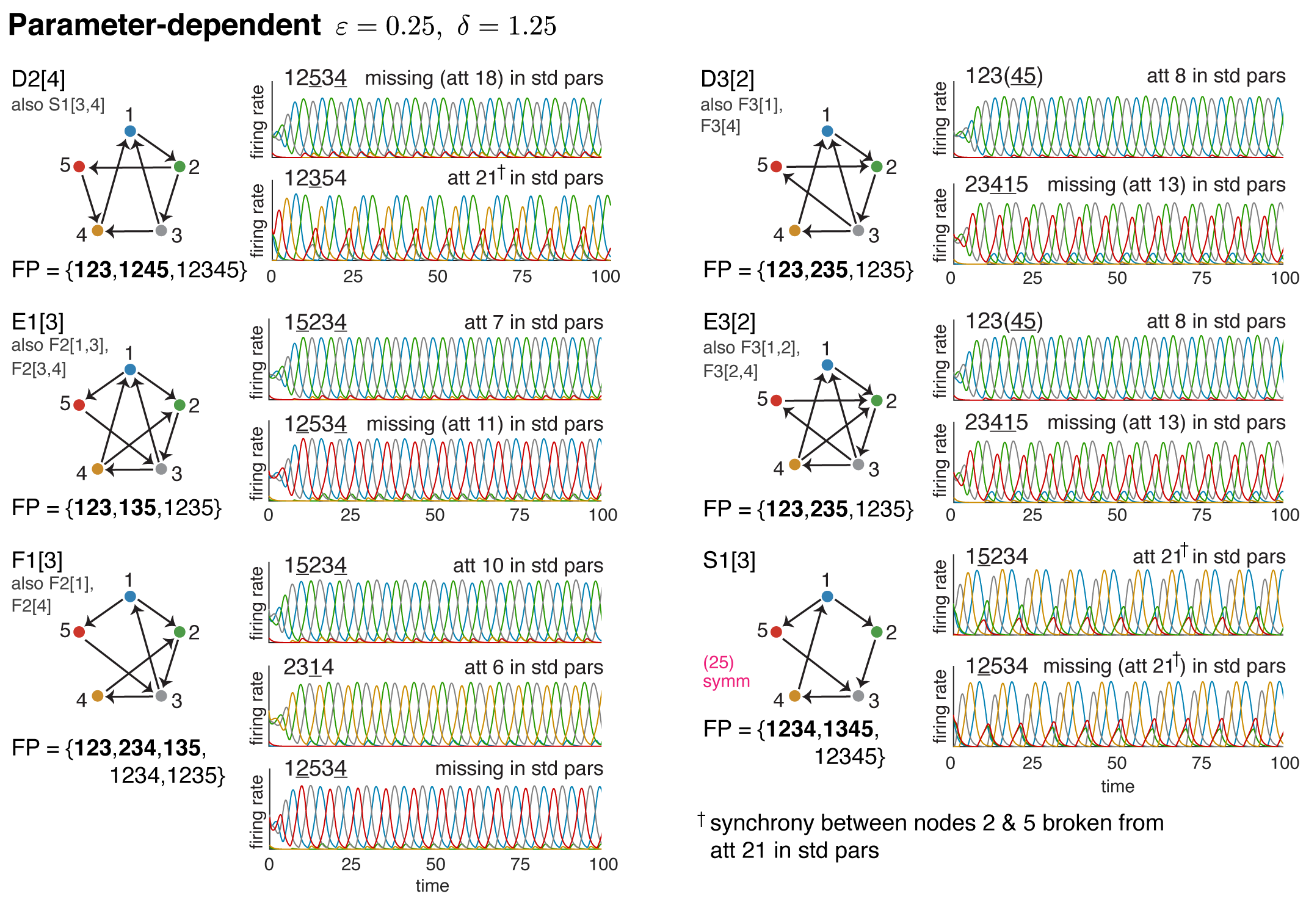

Supplement: S12 Fig — (TIF) [file pone.0264456.s012.tif]

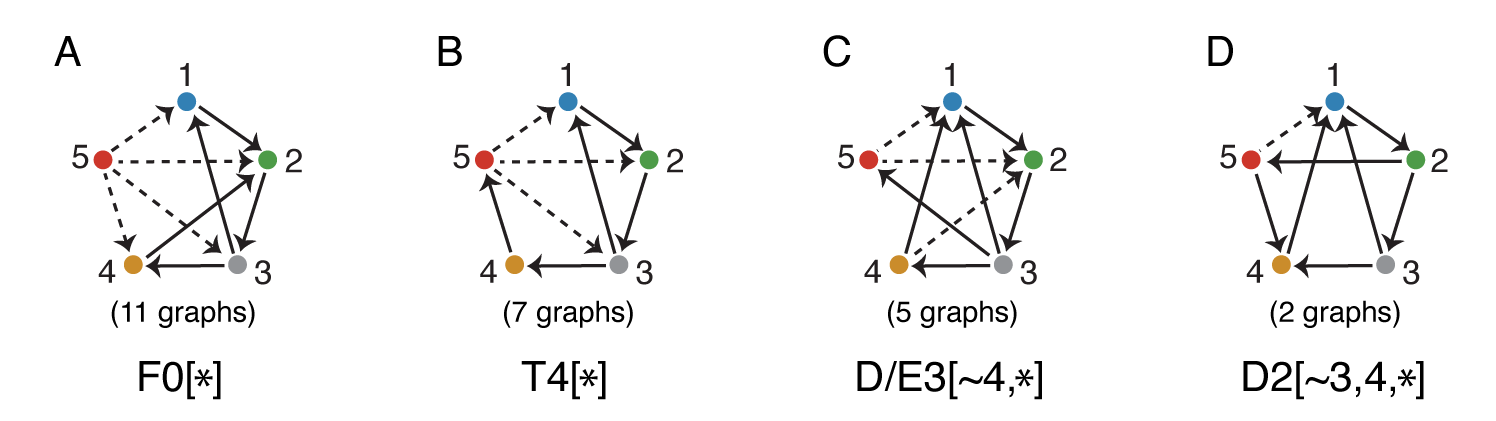

Supplement: S13 Fig — (TIF) [file pone.0264456.s013.tif]

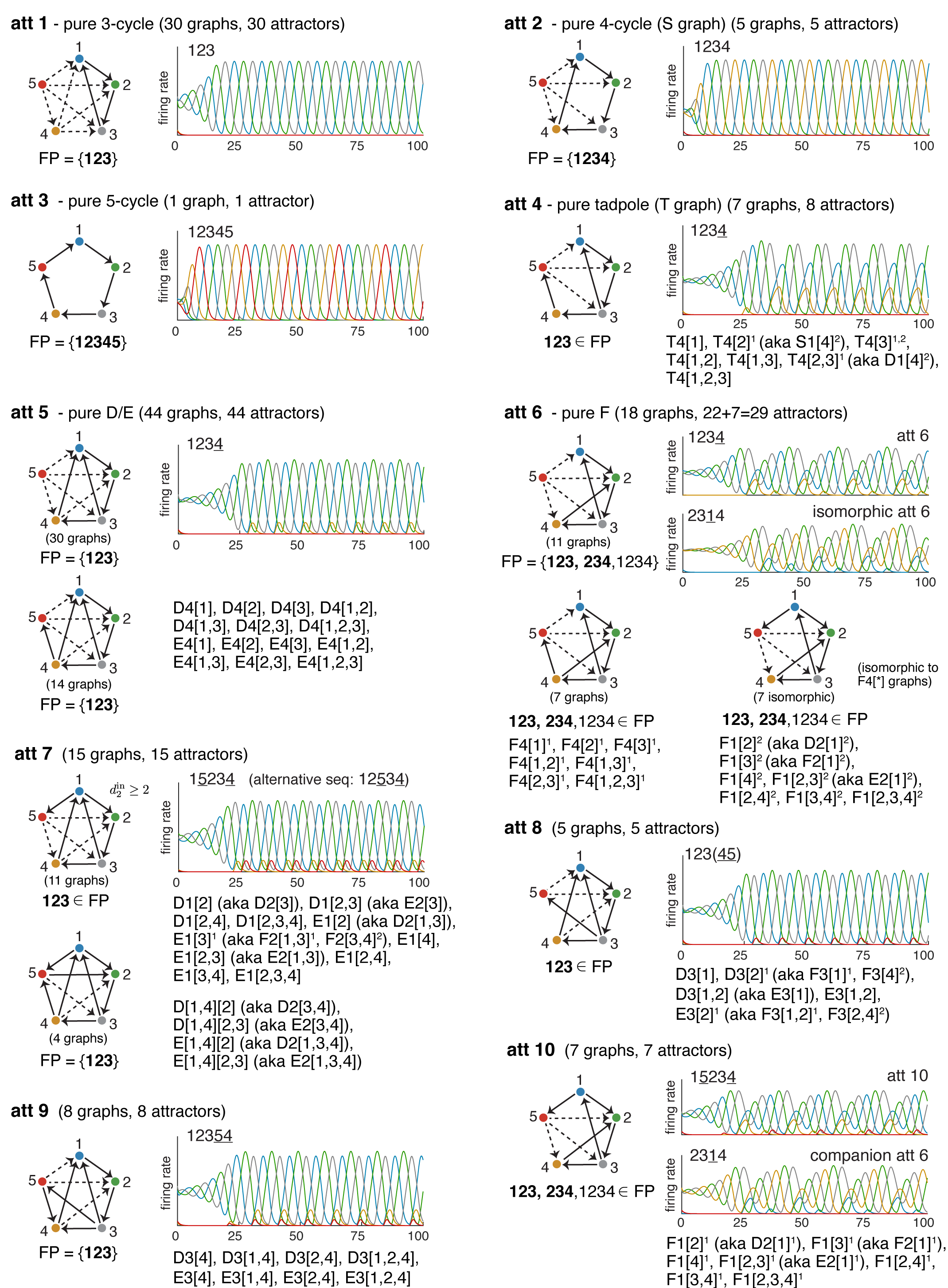

Supplement: S14 Fig — (TIF) [file pone.0264456.s014.tif]

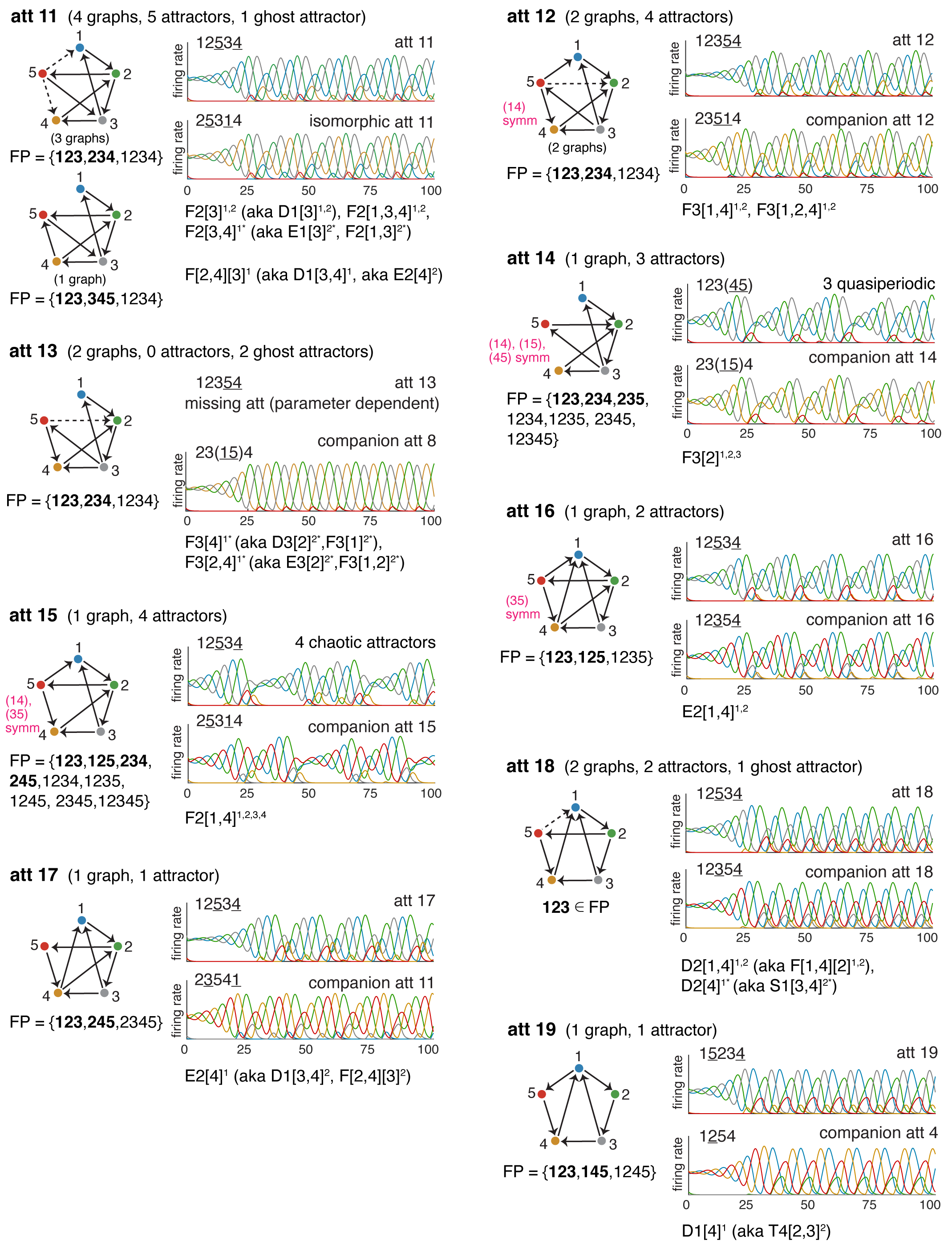

Supplement: S15 Fig — (TIF) [file pone.0264456.s015.tif]

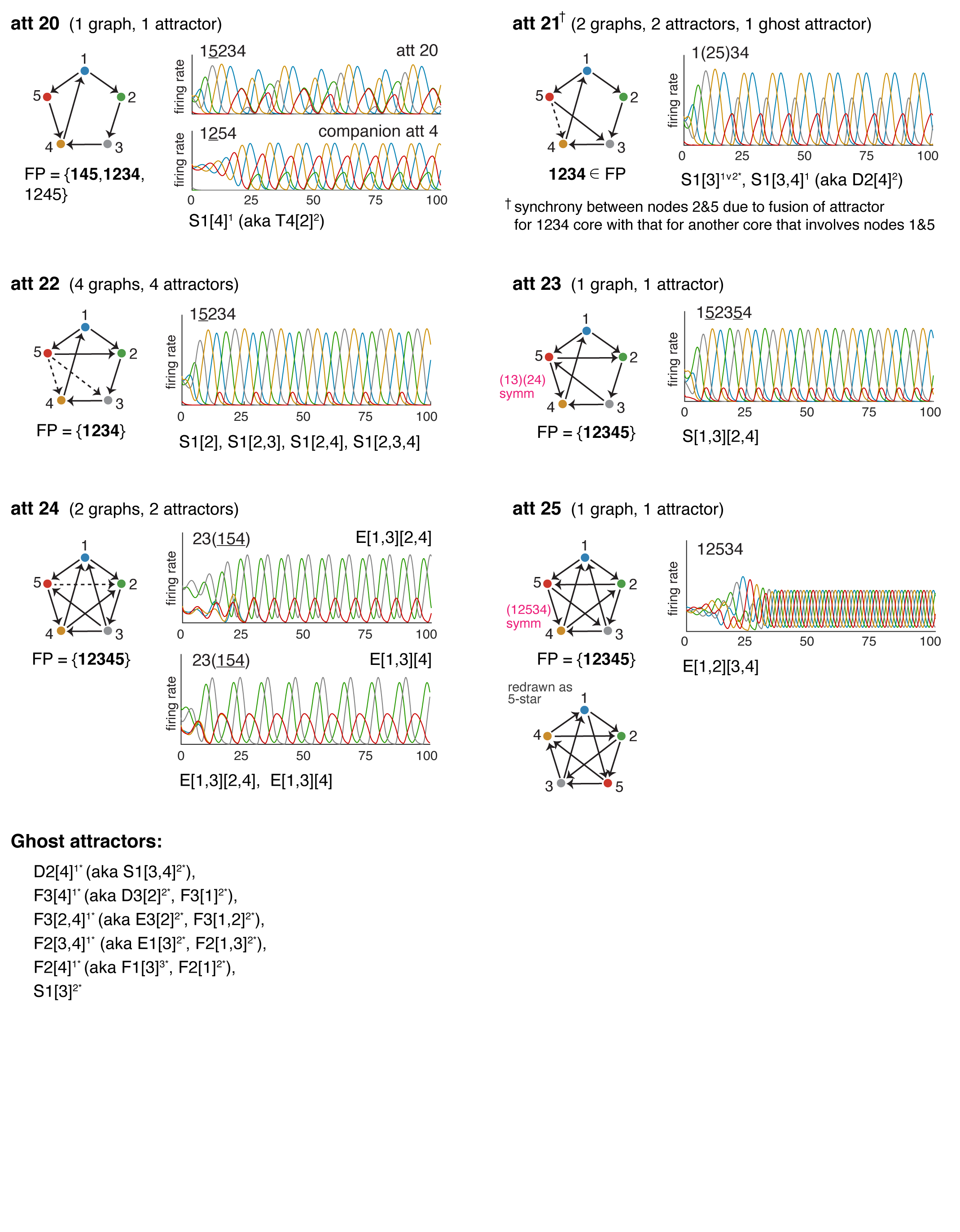

Supplement: S16 Fig — (TIF) [file pone.0264456.s016.tif]
