# Supplementary material for: Core motifs predict dynamic attractors in combinatorial threshold-linear networks
Source: PLoS One. 2022 Mar 4;17(3):e0264456. doi: 10.1371/journal.pone.0264456 (PMC8896682; doi:10.1371/journal.pone.0264456)
Supplement: S1 File — This supplementary text describes a classification scheme for oriented graphs with no sinks on n = 5 nodes. We also give a classification for all attractors of these graphs with standard parameters, and provide a dictionary of the attractor classes. (PDF) [file pone.0264456.s017.pdf]

Supporting Information

Taxonomy of attractors for oriented graphs on n=5 nodes

In this supplement we describe a classification scheme for oriented graphs with no sinks on $n = 5$ nodes and their attractors. Recall that a directed graph is *oriented* if it has no bidirectional edges, and it has *no sinks* if each node has at least one outgoing edge. We have chosen to study these graphs because their corresponding CTLNs are guaranteed to not have any stable fixed points [1, Theorem 2.4]. This enables us to focus our attention on testing the correspondence between core fixed points and dynamic attractors.

Our labeling method for oriented graphs relies on *base graphs* of smaller size. It aligns the vertices of core motifs that are embedded in similar ways. In particular, attractors stemming from the same core motif are aligned across graphs, so that their similarities and differences are more salient. Using this labeling scheme, we provide a dictionary of oriented $n = 5$ graphs, together with their corresponding attractors in the CTLN with standard parameters, $\varepsilon = 0.25$ and $\delta = 0.5$. This dictionary allowed us to classify the attractors based on sequence structure and visual similarity. The classification identified 25 distinct attractor classes from the 185 observed attractors. Moreover, we found that graphs supporting the same attractor clustered into highly structured graph families.

Contents

1 Base graphs and graph counts **1**

2 Constructed graphs and the labeling scheme **3**

3 Dictionary of n=5 oriented graphs with no sources or sinks **4**

4 Classification of dynamic attractors for $\varepsilon = 0.25, \delta = 0.5$ **17**

1. Base graphs and graph counts

The smallest oriented graph with no sinks is a 3-cycle. This implies that every oriented graph with no sinks on $n \leq 5$ nodes must be connected, since each connected component must have at least 3 nodes. We can split the $n = 5$ oriented graphs with no sinks into two parts: graphs with sources and graphs without any sources.

There are a total of 152 oriented graphs with no sinks on $n = 5$ nodes. We have verified that, with the exception of the 5-cycle, all of these graphs can be constructed by adding a single vertex to one of the five base graphs D, E, F, T, S, shown in Fig 1A, or two vertices to the 3-cycle. Since the D, E, F, and T bases cover all cases where there is an outgoing edge from the 3-cycle, the 3-cycle base is only needed for graphs where node 5 is a source, and node 4 is a source upon removal of 5. Note that the "tadpole" graph T has a sink node, 4, so any construction using this base must have a $4 \to 5$ edge.

In total, there are 76 graphs obtained by adding node 5 as a proper source[1] to one of the base graphs, 30 of them using the 3-cycle base (see Fig 1B). Note that we are counting non-isomorphic graphs, and symmetries make many of the counts nontrivial. When a base graph has no symmetry, as with the D and E graphs, there are $2^4 - 1 = 15$ distinct ways to add node 5 as a proper source (with at least one outgoing edge). On the other hand, when the base graph has symmetry, as in the F and S

[1] Recall that a *proper* source is a vertex with no incoming edges and at least one outgoing edge.

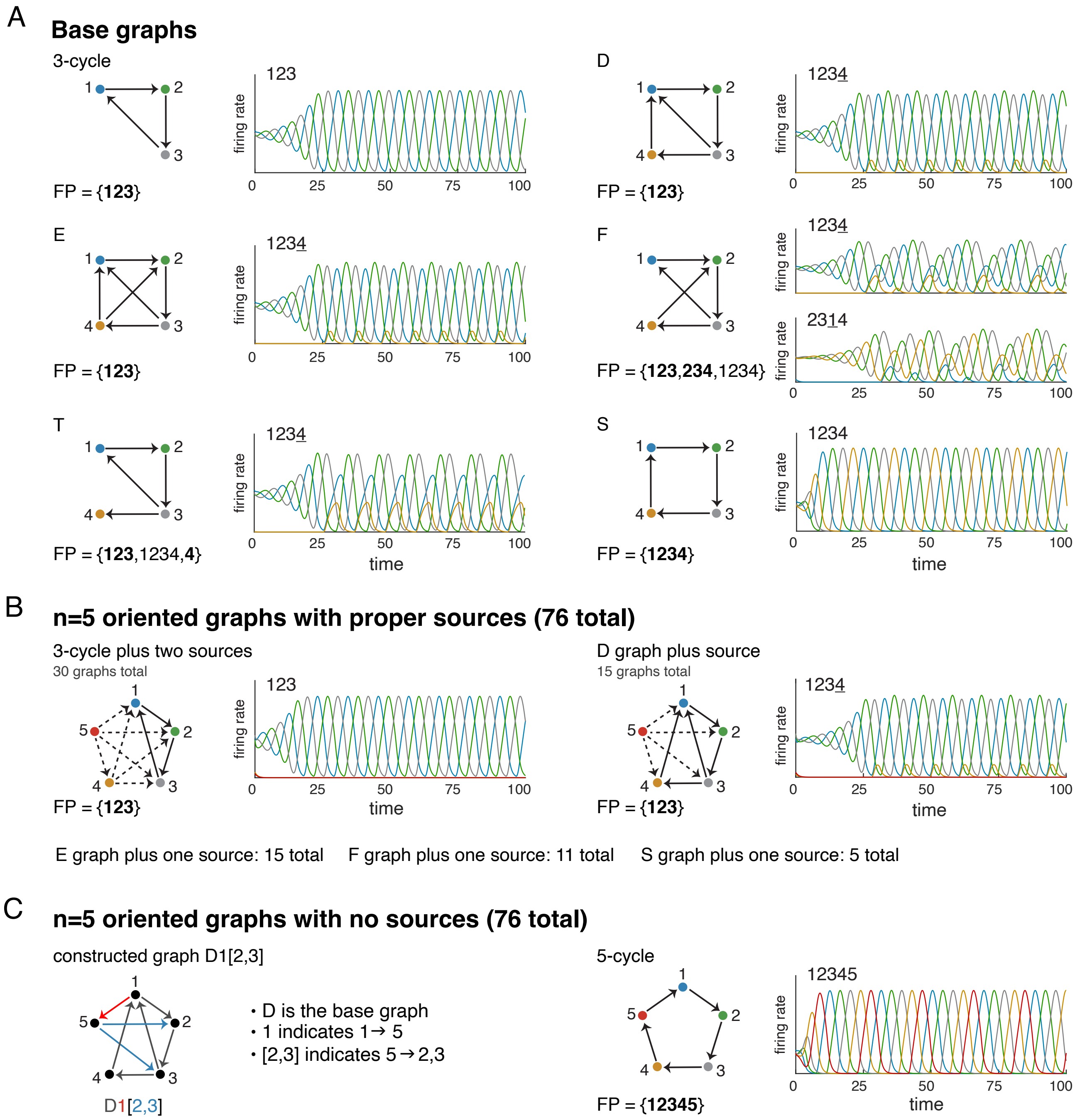

Figure 1: **Base graphs and graph counts.** (A) Base graphs used to construct $n = 5$ graphs, and their corresponding attractors. Each attractor has a sequence, indicating the (periodic) order in which the neurons achieve their peak firing rates. (B) The oriented graphs with sources can be constructed by adding proper sources to each of the base graphs. This yields 30 graphs from the 3-cycle base (left), 15 graphs from the D graph base (right), and an additional 15, 11, and 5 graphs from the E, F and S graph bases. (C) All oriented graphs with no sources or sinks can be constructed from one of the D, E, F, T, and S base graphs. (Left) For example, D1[2,3] is the graph constructed from the D graph with added edges $1 \to 5$ and $5 \to 2, 3$. (Right) The only oriented $n = 5$ graph with no sources or sinks that cannot be constructed in this way is the 5-cycle. (Same as Fig 6 in the main text.)

graphs, some of the choices for outgoing edges from the added node 5 are isomorphic, and the count is thus lower.

An additional 75 graphs constructed from a base graph do not have sources. Together with the 5-cycle, there are 76 oriented graphs with no sources or sinks (see Fig 1C). These are the graphs we focus

on for the labeling scheme and dictionary. Graphs with sources are simpler to classify: their attractors are identical to those of the corresponding base graphs in Fig 1A.

2. Constructed graphs and the labeling scheme

We have devised a labeling scheme for all the $n = 5$ graphs that can be constructed by adding a fifth node to one of the base graphs D, E, F, T, or S. We label each graph compactly with a letter and two sets of numbers indicating which nodes connect to 5 (see Fig 1C). The letter indicates the base graph, which is the induced subgraph on nodes 1 to 4. The number(s) immediately following the letter indicate nodes that send edges to 5; and the second set of numbers, in brackets, indicate nodes that receive edges from 5. Since these graphs are all oriented, a node cannot both send and receive an edge to 5, so the two sets of numbers are disjoint. They are also nonempty, since node 5 is neither a sink nor a source. Fig 2 illustrates the labeling scheme for the graphs D1[2,3] and S[1,3][2,4]. Fig 3 depicts how, given a graph, one can find its label(s).

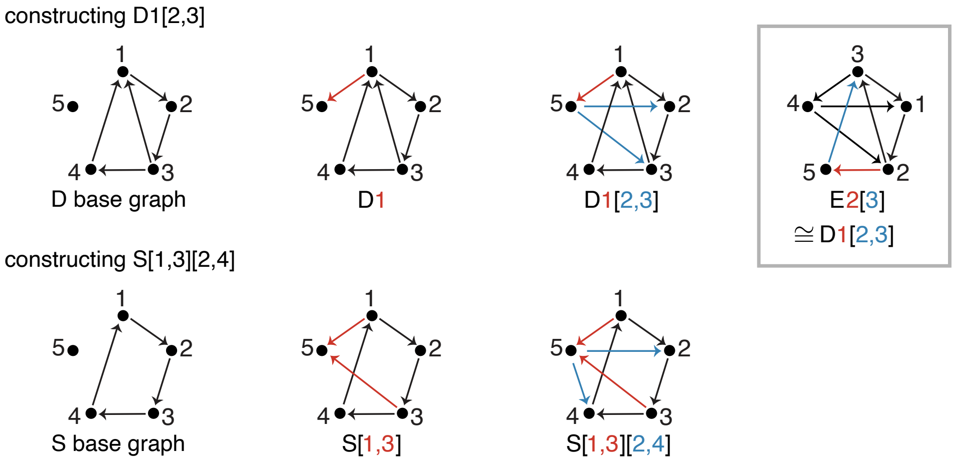

Figure 2: **Construction of oriented graphs from base graphs.** (Top) Starting with a D base, the graph D1[2,3] is constructed by adding a node 5 together with incoming edge $1 \to 5$ (red) and outgoing edges $5 \to 2$ and $5 \to 3$ (blue). An isomorphic graph, E2[3], can be constructed from an E base. (Bottom) The graph S[1,3][2,4] has two incoming edges to node 5, given in the first set of brackets. This graph cannot be constructed from any base with only one edge into node 5.

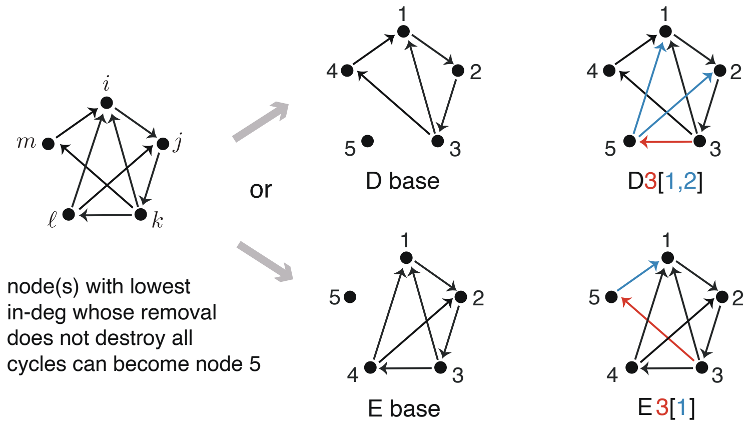

Figure 3: **Finding the name for a given oriented graph with no sinks.** The nodes with lowest in-degree are k, ℓ, and m. However, removing k results in a graph with no cycles that cannot match one of our base graphs. Removing ℓ (top) uncovers a D graph base, while removing m (bottom) results in an E base. The original graph can thus be labeled as D3[1,2] or E3[1].

Notice that constructions with different bases can produce isomorphic graphs. For example, we see that D1[2,3] $\cong$ E2[3] in Fig 2 and D3[1,2] $\cong$ E3[1] in Fig 3, under appropriate relabelings of the nodes.

In order to maximally align our attractors and take advantage of the common base graph structures, we prioritize representations that minimize the number of edges to node 5. It turns out that a labeling where node 5 has in-degree 0 or 1 exists for all but four graphs: E[1,2][3,4], E[1,3][2,4], E[1,3][4], and S[1,3][2,4].[2] We can also eliminate some redundancy for constructions with an F graph. Since F has a (1,4) exchange symmetry, it follows that graphs of the form F1[$*$] are isomorphic to graphs of the form F4[$*$]. Similarly, F2[1] is isomorphic to F2[4]. In our dictionary of graphs, we choose 1 over 4 whenever possible.

With these restrictions, our construction method produces 91 oriented graphs with no sinks and no sources (excluding the 5-cycle). We have not eliminated all redundancy, however, and 16 of these graphs appear twice under two different names. Our construction thus accurately recovers all 75 non-isomorphic oriented graphs with no sinks and no sources, other than the 5-cycle.

Table 1 displays families of constructed graphs, with and without sources, organized by base graph. Every oriented $n = 5$ graph with no sinks is covered by one (or more) of these families. The notation for graph families works as follows. The families with a "0" in the label, D0[$*$], E0[$*$], etc., indicate that a source was added to the base graph. The [$*$] indicates that edges out of node 5 are all optional, and can occur in any possible combination provided at least one is selected. Note that T0[$*$] consists of graphs with node 4 as a sink, which do not belong to our oriented graphs with no sinks family. We include it because it is the first graph family where att 4 emerges, but it does not count towards the total number of graphs with sources.

The remaining families have labels such as D2[$*$] and E[1,3][4,$*$], which consists of graphs that do not have sources. The graph family D2[$*$] consists of graphs with the $2 \to 5$ edge, with the outgoing edges $5 \to 1, 3, 4$ all optional. It is understood that $5 \not\to 2$ because there can be no bidirectional edges, while at least one outgoing edge must be selected since 5 is not a sink. In the case of E[1,3][4,$*$], the $*$ indicates that $5 \to 2$ is optional, as that is the only possibility left.

For each graph family in Table 1, the number of graphs is easy to count when one takes symmetry into account and recalls that node 5 must have at least one outgoing edge. Note also that there is no F4[$*$] family listed, since it is isomorphic to F1[$*$]. Similarly, there are no S2[$*$], S3[$*$], or S4[$*$] families, since they are all isomorphic to S1[$*$]. Finally, T4[$*$] is the only T graph family without sinks, since we must have the $4 \to 5$ edge whenever T is the base graph.

The attractor classes in Table 1 were defined by first enumerating the distinct attractors arising in the base graphs, together with the 5-cycle. Note that the D and E graphs have the same attractor (see Fig 1A). The remaining attractor classes were defined from the dictionary, given in the next section, by introducing a new number for each new type of attractor that arose.

3. Dictionary of n=5 oriented graphs with no sources or sinks

It follows from the *sources graph rule* (see Table 1 of the main text) that proper sources cannot participate in fixed point supports. Moreover, it was proven in [2] that if i is a proper source in G, then $\mathrm{FP}(G) = \mathrm{FP}(G|_{[n]\setminus i})$. In other words, proper sources can be iteratively removed from a graph without altering the fixed points of the corresponding CTLN. This means that for graphs obtained by adding a source node to one of the base graphs, the set of fixed point supports $\mathrm{FP}(G)$ exactly matches that of the base. As it turns out, the attractors in the S0[$*$], T0[$*$], D0[$*$], E0[$*$], F0[$*$], and 3-cycle + sources families also exactly match the attractors of the corresponding base graphs, which are shown in Fig 1A. Together with the 5-cycle, this covers the first seven lines of Table 1.

The remaining 92 graphs have no sources or sinks, and all but the 5-cycle can be constructed from one of the base graphs D, E, F, T, and S. We have already seen the attractor for the 5-cycle in Fig 1C. In

[2] In these four graphs, the core motif of the base graph does not survive the addition of node 5, thus producing a new core motif of size $n = 5$.

graph family	# graphs	attractor classes
3-cycle + sources	30	att 1
4-cycle + source (aka S0[∗])	5	att 2
5-cycle (no source) – core motif	1	att 3
T0[∗] (note: all have sinks!)	15	att 4
D0[∗]	15	att 5
E0[∗]	15	att 5
F0[∗]	11	att 6
D1[∗]	7	att 4, 7, 11, 17, 19
D2[∗]	7	att 6, 7, 10, 18, 21
D3[∗]	7	att 8, 9
D4[∗]	7	att 5
E1[∗]	7	att 7
E2[∗]	7	att 6, 7, 10, 11, 16, 17
E3[∗]	7	att 8, 9
E4[∗]	7	att 5
F1[∗]	7	att 6, 10
F2[∗]	5	att 6, 7, 10, 11, 15
F3[∗]	5	att 8, 12, 14
T4[∗]	7	att 4, 19, 20
S1[∗]	7	att 4, 20, 21, 22
S[1,3][2,4] – core motif	1	att 23
E[1,3][4,∗] – core motifs	2	att 24
E[1,2][3,4] – core motif	1	att 25
total with sources (excluding T0[∗])	76	att 1, 2, 5, 6
total with no sources (includes duplicates)	92	atts 3–25
total with no sources – non-isomorphic	76	atts 3–25

Table 1: Graph families organized by base graph.

the remainder of this section, we provide a complete dictionary for the 91 graphs in Table 1, starting with graph family D1[∗] (line 8) and ending with E[1,2][3,4] (line 23). Some of these are isomorphic copies of the same graph, with different base graphs corresponding to different permutations of the nodes. It is useful to view these in both representations, however, as they can "center" the graph on different core fixed points. In particular, most of the graphs with multiple attractors have more than one base graph representation. Note, however, that in the case of the F graphs we do not include isomorphic copies that stem from the obvious (1,4) exchange symmetry. In particular, we do not include F4[∗] graphs in our dictionary, just as they were not included in Table 1. We also remove isomorphic copies from the F2[∗] and F3[∗] families that are due to the (1,4) symmetry, which is why these families have only 5 graphs each, as opposed to 7.

Our dictionary consists of eight pages of graphs, two for each of the D, E, and F graph families, one for the S and T families (combined), and one for the constructed core motifs: S[1,3][2,4], E[1,3][4], E[1,3][2,4], and E[1,2][3,4], which appear in the bottom three rows of Table 1. (Note that the core motifs must all have two incoming edges to node 5, as the added node must "kill" the core fixed point for the cycle in the base graph.)

From [2, Theorem 7], we know that $\mathrm{FP}(G)$ for any oriented graph on $n \leq 5$ nodes is parameter-independent, provided ε and δ are in the legal range. For each graph, we were thus able to compute

$\mathrm{FP}(G)$ using graph rules. Furthermore, we identified the core fixed points, which all have minimal support. Their supports are indicated in bold.

We then simulated solutions to the corresponding CTLN with standard parameters, $\varepsilon = 0.25$ and $\delta = 0.5$, and systematically searched for attractors using a battery of dozens of initial conditions, the same for all graphs. To this we added initial conditions that were perturbations from each fixed point (not only core fixed points). We plotted the resulting solutions up to time 100 in units of the leak timescale, τ, which has been normalized to 1 in our TLN equations. All solutions converged quickly to an attractor in this time. Moreover, each observed attractor could be accessed via an initial condition that was a perturbation of a core fixed point. In the following dictionary pages, we selected example solutions obtained from this set, so that the transient activity shows how the activity spirals away from the core fixed point before converging to the attractor. All observed attractors are displayed next to each graph. In the case of F3[2] and F2[1,4], there are three and four isomorphic attractors, respectively. Here we showed only two examples of each, as the rest could be inferred by symmetry.

We classified attractors systematically as follows: for each new graph, we examined all attractors and compared them to the previously observed attractor classes. This often entailed permuting the variables, $x_1(t), \ldots, x_5(t)$, until two attractors were maximally aligned. We examined firing rate curves, as shown in the following dictionary pages, and also random projections of the trajectories in $\mathbb{R}^5$. Only when agreement between two attractors was near perfect did we decide to cluster them into the same class. Altogether, we observed 97 attractors across the 75 non-isomorphic oriented graphs listed in our dictionary pages. While some of them were repeats of attractor classes 4-6, we also identified 19 new classes. After reordering these classes to give nearby numbers to similar attractors, we came up with final names for them as att 7-25. These attractor labels are shown in the upper right corner of each firing rate plot.

Note that secondary attractors superficially look different (e.g., the colors on the rate curves don't match) because they are centered on a different permutation, where the core fixed point is not supported on 123 or 1234. Moreover, even when the core fixed points have the same support, attractors may not be fully aligned. For example, D1[2] and D2[3] are isomorphic and each have a single core fixed point supported on 123. The attractors have different sequences, though, which may lead one to believe they are distinct. However, there is a permutation that takes the labeled graph for D2[3] to D1[2], and this permutation reveals that the attractors are in fact identical and have the same sequence (as expected, since the graphs are isomorphic). Similarly, there is a permutation that realigns the attractor for D2[1,3], as it has the same sequence as D2[3]. All three of these graphs have the same attractor, att 7.

Altogether, across the 75 non-isomorphic graphs in our dictionary, there were 103 core fixed points. We thus predicted 103 dynamic attractors. However, we only observed 97 attractors.[3] Although every observed attractor was predicted by a core fixed point, there were 6 instances where a core fixed point did not have a corresponding attractor. We refer to these as *ghost attractors*, because they failed to be realized in the standard parameters. Each graph that has a ghost attractor is labeled with a $**$ in the dictionary, and the core fixed point with the missing attractor has a superscript *. For example, on the first page, the graph D2[4] has a ghost attractor corresponding to the core fixed point with support 123. At the end of this section, we return to these ghost attractors and show that they can be realized by CTLNs in a higher δ parameter regime.

The vast majority of graphs have only one or two core fixed points, with at most two dynamic attractors that are limit cycles. Only three graphs have more than two core fixed points. They are each somewhat exotic:

- F3[2] has 3 core fixed points and 3 quasiperiodic attractors, all isomorphic to each other.

[3]When comparing to Table 2 in the main text, recall that the "with no sources" line in the table includes the 5-cycle, so it has one extra graph, core fixed point, and attractor.

- F2[1,4] has 4 core fixed points and 4 chaotic attractors, all isomorphic to each other.
- F1[3] has 3 core fixed points, but one leads to a ghost attractor. This graph has three 3-cycles, but they are not symmetrically embedded. The 123 and 234 cycles yield distinct attractors, while the 135 cycle corresponds to a ghost attractor.

Finally, for graphs that have nontrivial automorphism groups we indicate the symmetries in pink.

D graphs: D1[∗] & D2[∗]

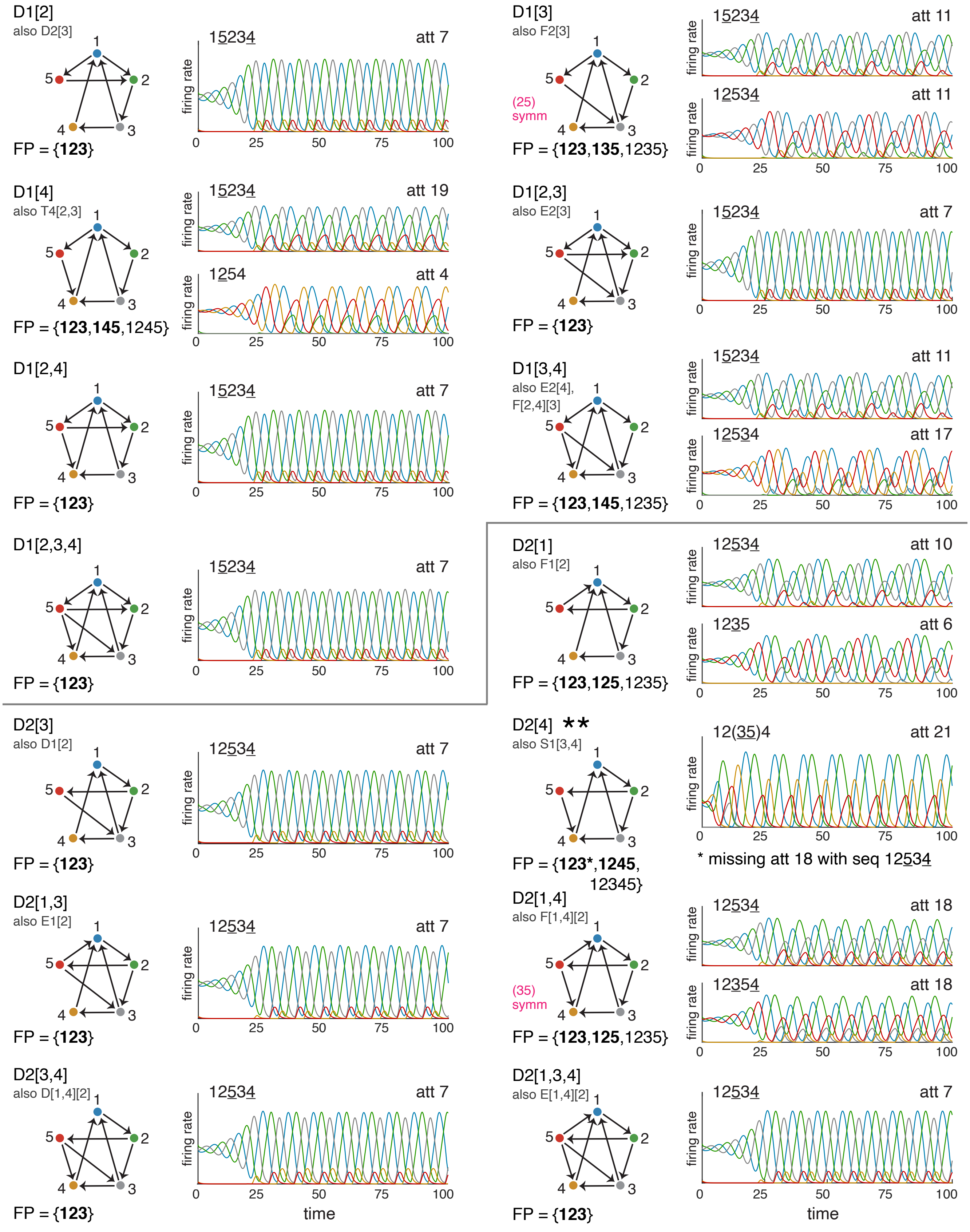

D graphs: D3[∗] & D4[∗]

D3[1]

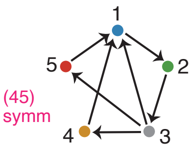

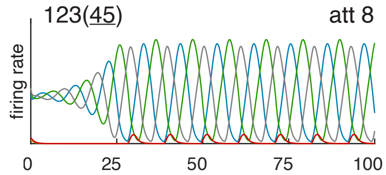

FP = {**123**}

D3[2] ⋆⋆

also F3[1], F3[4]

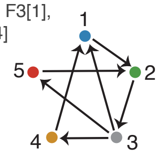

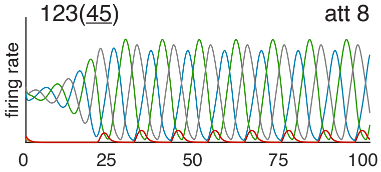

FP = {**123**,**235***,1235}

* missing att 13 with seq 23415

D3[4]

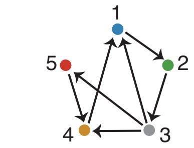

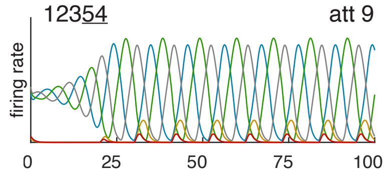

FP = {**123**}

D3[1,2]

also E3[1]

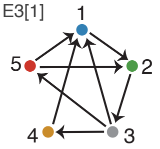

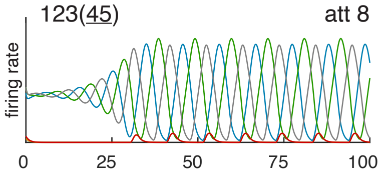

FP = {**123**}

D3[1,4]

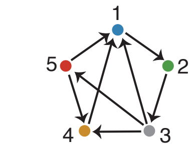

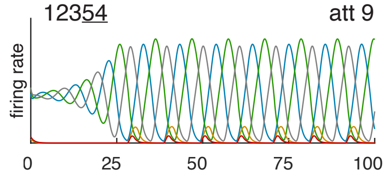

FP = {**123**}

D3[2,4]

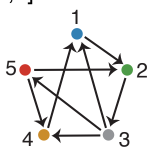

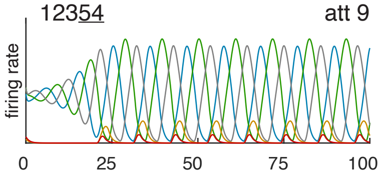

FP = {**123**}

D3[1,2,4]

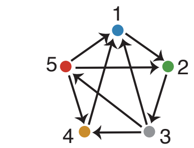

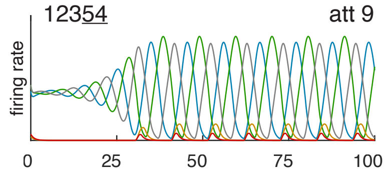

FP = {**123**}

D4[1]

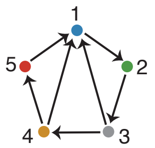

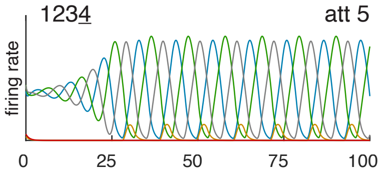

FP = {**123**}

D4[2]

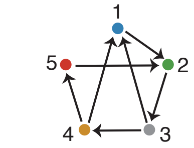

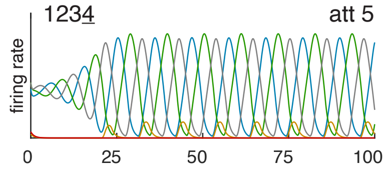

FP = {**123**}

D4[3]

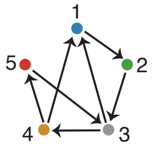

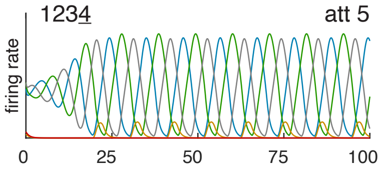

FP = {**123**}

D4[1,2]

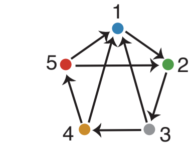

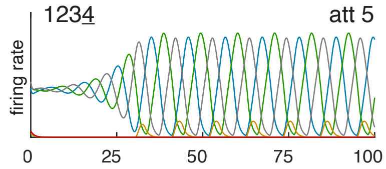

FP = {**123**}

D4[1,3]

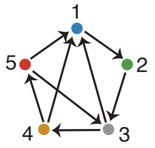

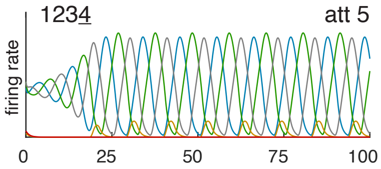

FP = {**123**}

D4[2,3]

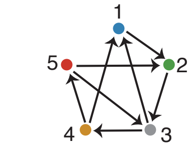

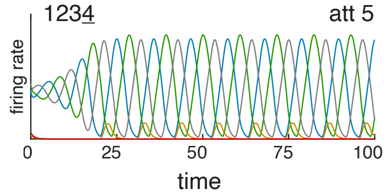

FP = {**123**}

D4[1,2,3]

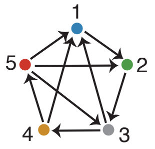

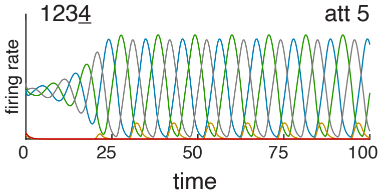

FP = {**123**}

E graphs: E1[∗] & E2[∗]

E1[2]
also D2[1,3]

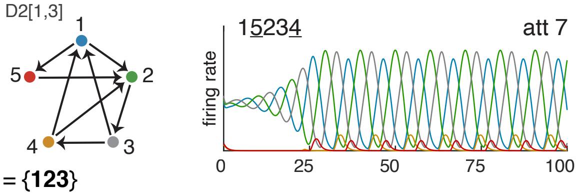

FP = {**123**}

E1[3] ⋆⋆
also F2[1,3]
F2[3,4]

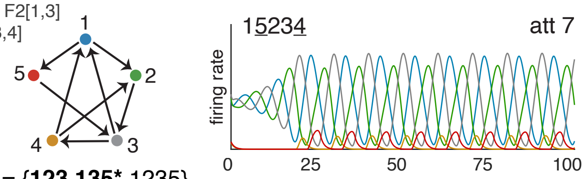

FP = {**123**,**135***,1235}

* missing att 11 with seq 12534

E1[4]

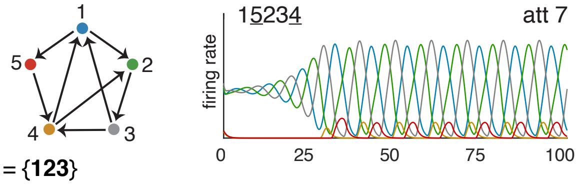

FP = {**123**}

E1[2,3]
also E2[1,3]

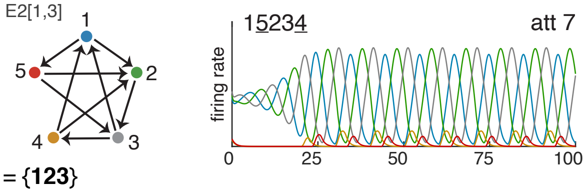

FP = {**123**}

E1[2,4]

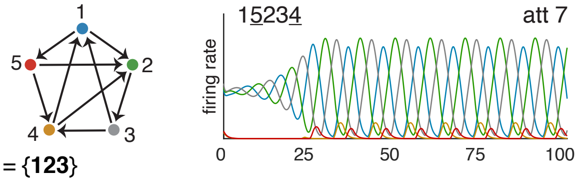

FP = {**123**}

E1[3,4]

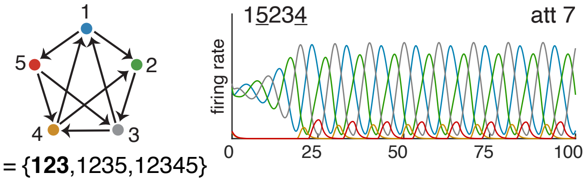

FP = {**123**,1235,12345}

E1[2,3,4]

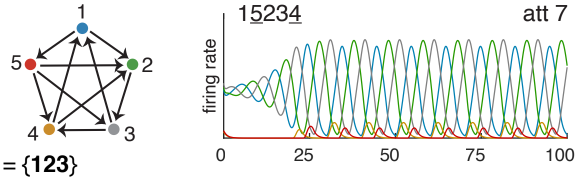

FP = {**123**}

E2[1]
also F1[2,3]

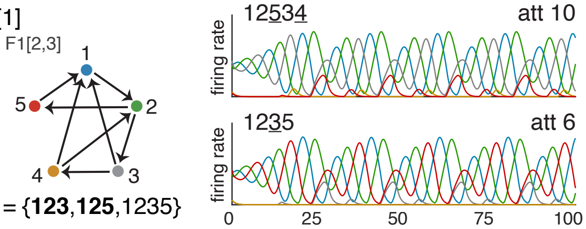

FP = {**123**,**125**,1235}

E2[3]
also D1[2,3]

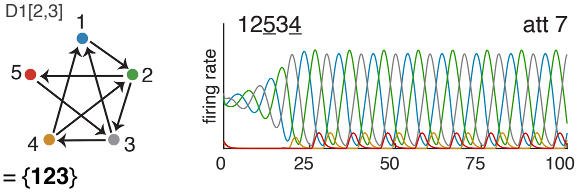

FP = {**123**}

E2[4]
also D1[3,4],
F[2,4][3]

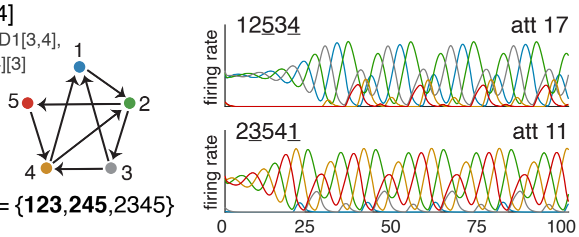

FP = {**123**,**245**,2345}

E2[1,3]
also E1[2,3]

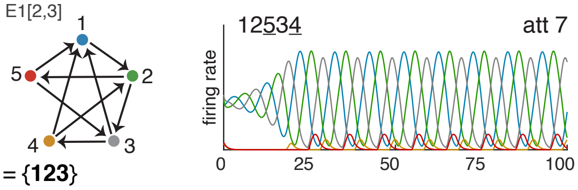

FP = {**123**}

E2[1,4]

(35)
symm

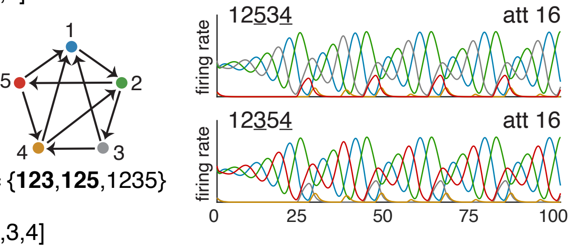

FP = {**123**,**125**,1235}

E2[3,4]
also D[1,4][2,3]

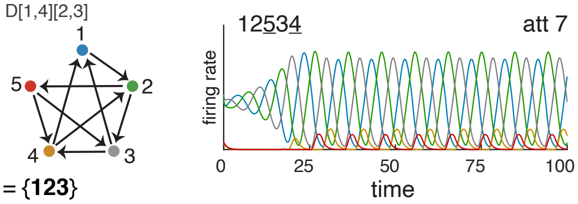

FP = {**123**}

E2[1,3,4]
also E[1,4][2,3]

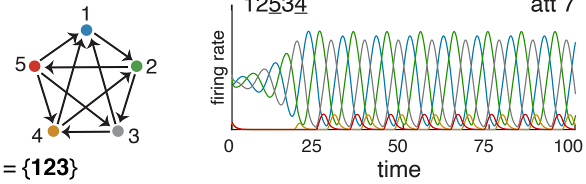

FP = {**123**}

E graphs: E3[∗] & E4[∗]

E3[1]
also D3[1,2]

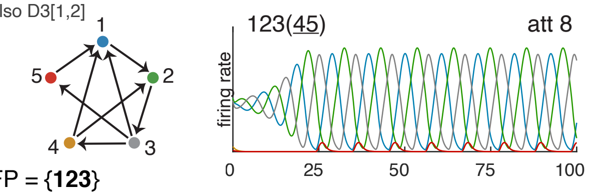

FP = {**123**}

E3[2] ⋆⋆
also F3[1,2], F3[2,4]

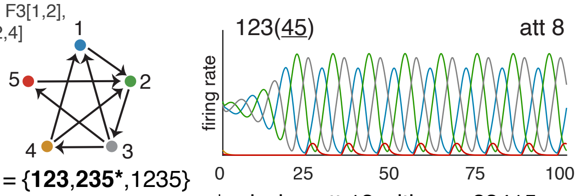

FP = {**123**,**235***,1235}

* missing att 13 with seq 23415

E3[4]

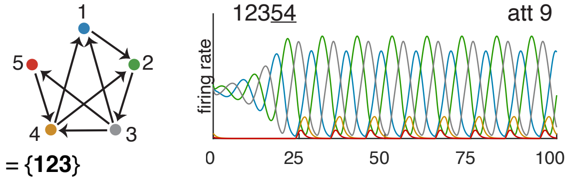

FP = {**123**}

E3[1,2]

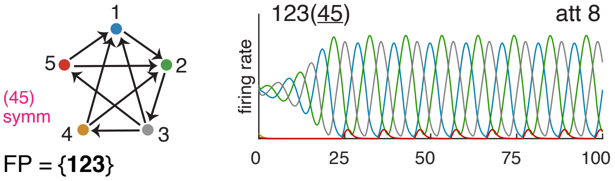

FP = {**123**}

E3[1,4]

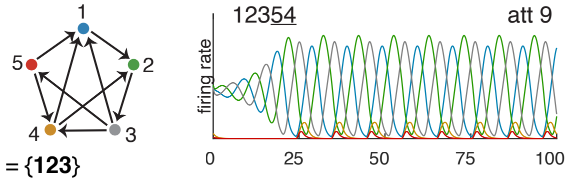

FP = {**123**}

E3[2,4]

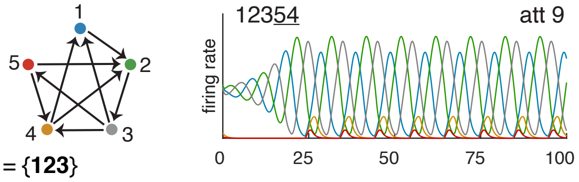

FP = {**123**}

E3[1,2,4]

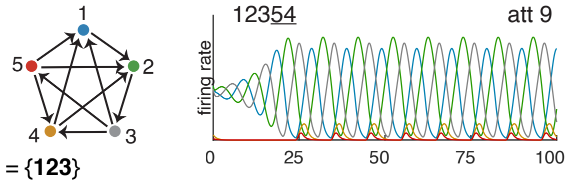

FP = {**123**}

E4[1]

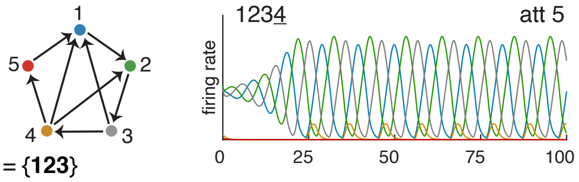

FP = {**123**}

E4[2]

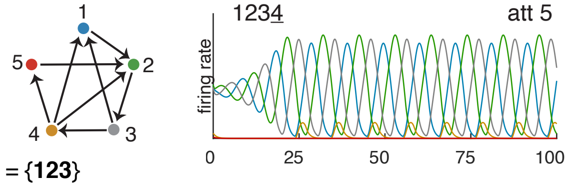

FP = {**123**}

E4[3]

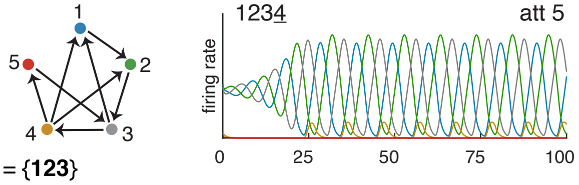

FP = {**123**}

E4[1,2]

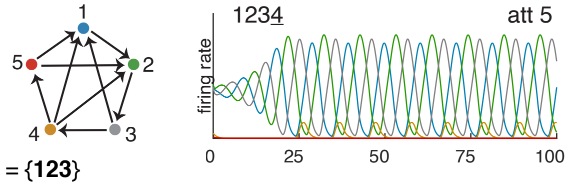

FP = {**123**}

E4[1,3]

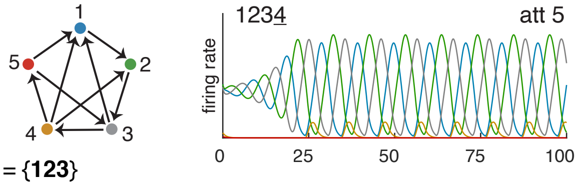

FP = {**123**}

E4[2,3]

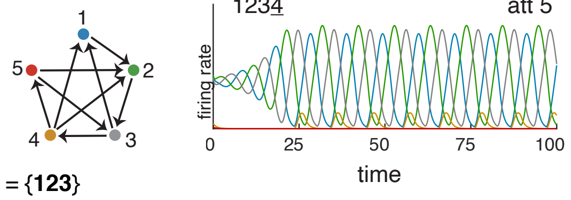

FP = {**123**}

E4[1,2,3]

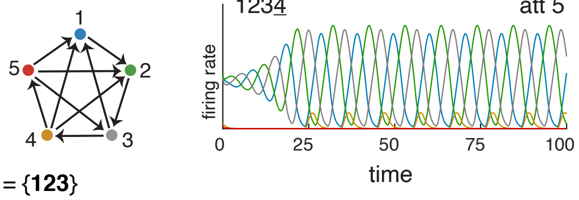

FP = {**123**}

F graphs: F1[∗] & F2[∗]

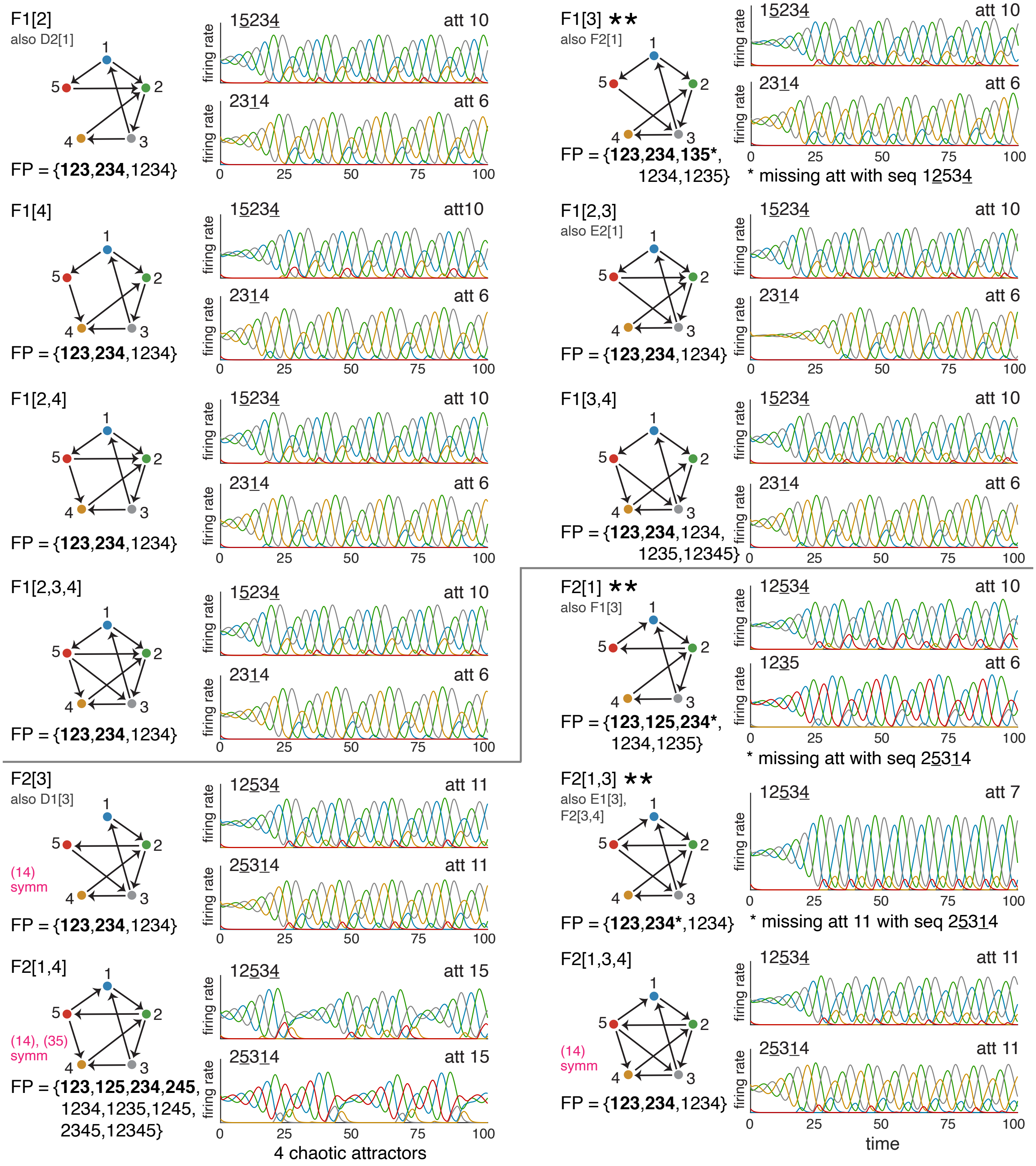

F graphs: F3[∗]

F3[1] ★★

also D3[2], F3[4]

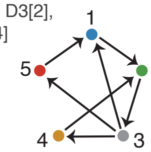

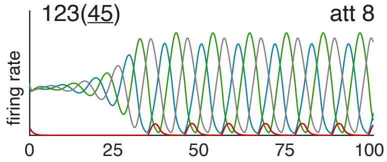

FP = {**123**,**234***,1234}

* missing att 13 with seq 23514

F3[1,2] ★★

also E3[2], F3[2,4]

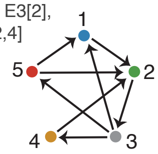

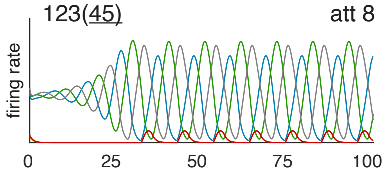

FP = {**123**,**234***,1234}

* missing att 13 with seq 23514

F3[1,2,4]

(14) symm

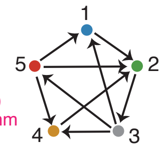

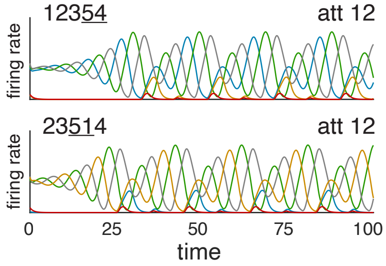

FP = {**123**,**234**,1234}

F3[2]

(14), (15), (45) symm

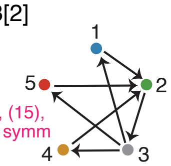

FP = {**123**,**234**,**235**, 1234,1235, 2345,12345}

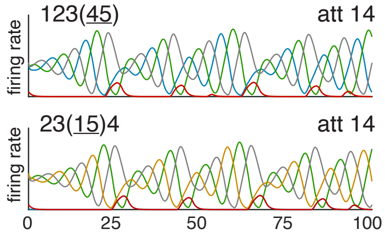

3 quasiperiodic

F3[1,4]

(14) symm

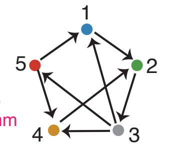

FP = {**123**,**234**,1234}

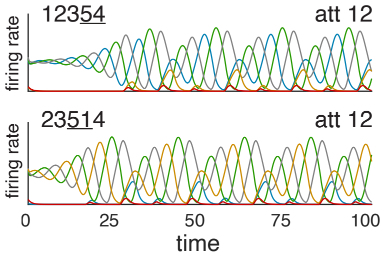

T and S graphs: T4[∗] & S1[∗]

T4[1]

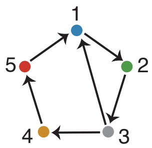

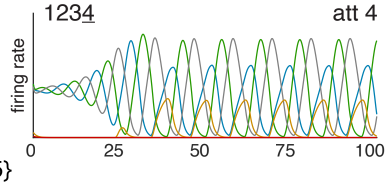

FP = {**123**,1234,12345}

T4[2]
also S1[4]

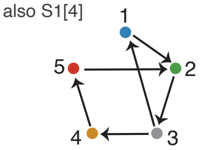

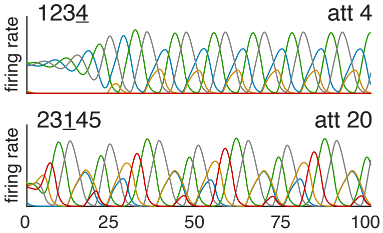

FP = {**123**,1234,**2345**}

T4[3]

(14)(25)
symm

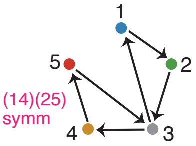

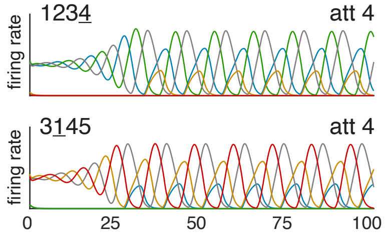

FP = {**123**,**345**,1234,
1345,12345}

T4[1,2]

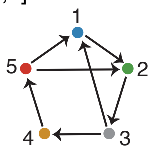

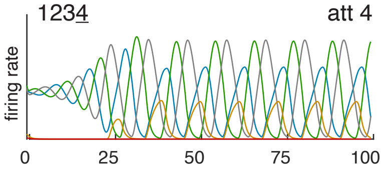

FP = {**123**,1234,12345}

T4[1,3]

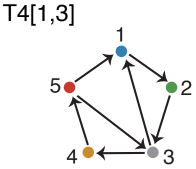

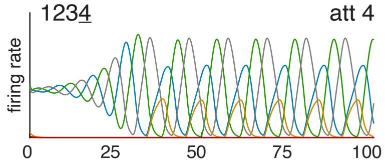

FP = {**123**,1234,12345}

T4[2,3]
also D1[4]

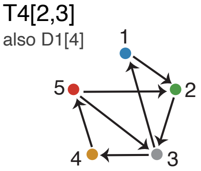

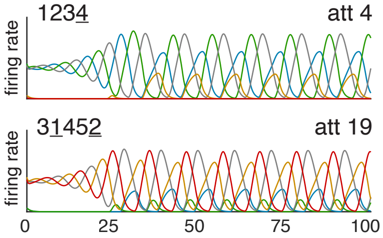

FP = {**123**,**345**,1234}

T4[1,2,3]

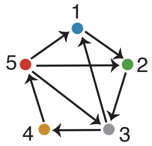

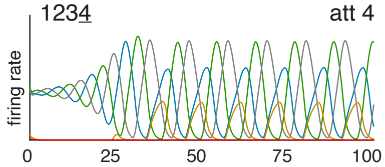

FP = {**123**,1234,12345}

S1[2]

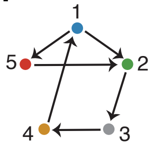

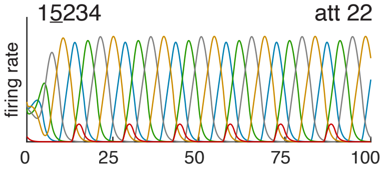

FP = {**1234**}

S1[3] ⋆⋆

(25)
symm

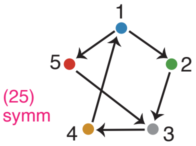

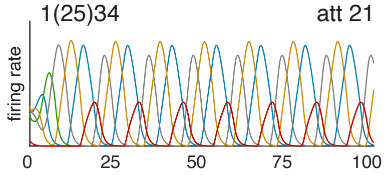

FP = {**1234***,**1345***,
12345}

* missing att 21 with seq 12534

S1[4]
also T4[2]

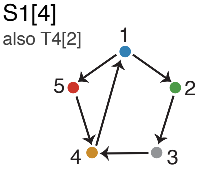

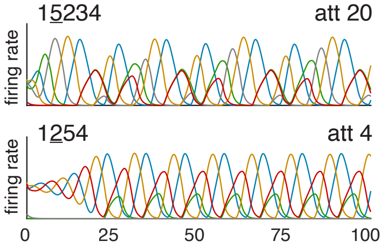

FP = {**1234**,**145**,
1245}

S1[2,3]

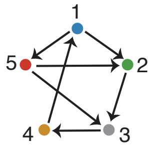

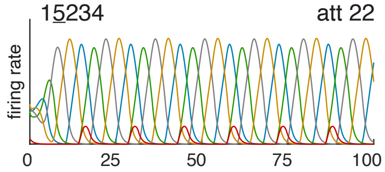

FP = {**1234**}

S1[2,4]

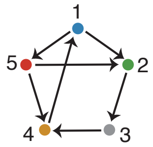

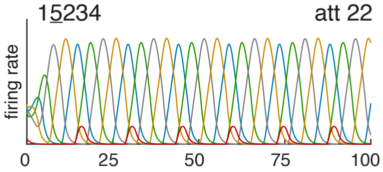

FP = {**1234**}

S1[3,4] ⋆⋆
also D2[4]

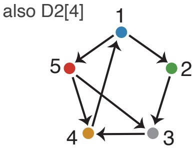

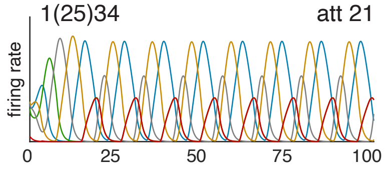

FP = {**1234**,**145***,
12345}

* missing att 18 with seq 12534

S1[2,3,4]

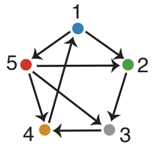

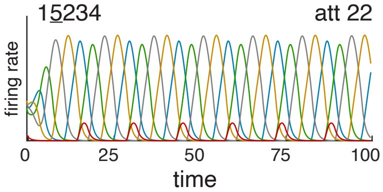

FP = {**1234**}

Oriented $n = 5$ core motifs

Recall that the 5-cycle is the only oriented graph on $n = 5$ nodes with no sources or sinks that cannot be constructed from one of the D, E, F, T or S base graphs. It also happens to be a core motif. But there are four additional $n = 5$ core motifs, and these can all be constructed from the base graphs. However, they each require two incoming edges to the fifth node, since the added node must eliminate the core fixed point from the cycle in the base graph. Together, they comprise the last three graph families in Table 1. Note that E[1,2][3,4], drawn here to make apparent the cyclic symmetry, has an additional spurious attractor in the non-standard parameters $\varepsilon = 0.1, \delta = 0.12$.

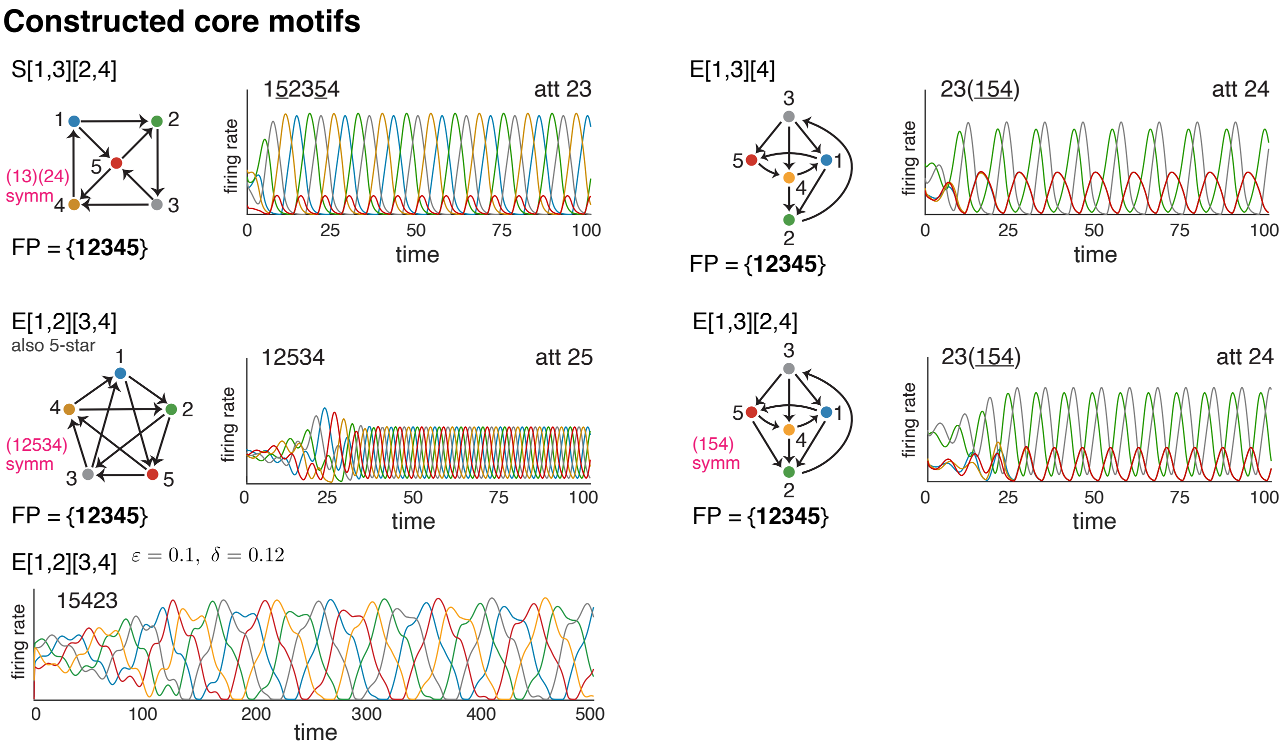

Graphs with ghost attractors, revisited

Table 2 summarizes the total numbers of core fixed points and attractors for $n = 5$ oriented graphs with and without sources.

graphs	# graphs	# core fps	# attractors	# ghost atts	# spurious atts
with a source	76	87	87	0	0
with no source	76	104	98	6	0
total	152	191	185	6	0

Table 2: Core fixed points and attractors for $n = 5$ oriented graphs with no sinks. The attractors were found in CTLNs with the standard parameters, $\varepsilon = 0.25, \delta = 0.5$.

Note that there are 6 core fixed points that did not exhibit a corresponding attractor. Here we see that these so-called "ghost" attractors can be recovered as realizable attractors in a different parameter regime. Specifically, if we simulate CTLNs with a significantly higher δ, causing the strong inhibition to be stronger, we end up obtaining a distinct attractor for each of the core fixed points in the problematic graphs. In the figure below, the "ghost" attractors are labeled as "missing in standard parameters." Moreover, by comparing the attractors across graphs in this parameter regime, we were able to identify attractor classes for all but one of the ghosts.

Parameter-dependent $\varepsilon = 0.25,\ \delta = 1.25$

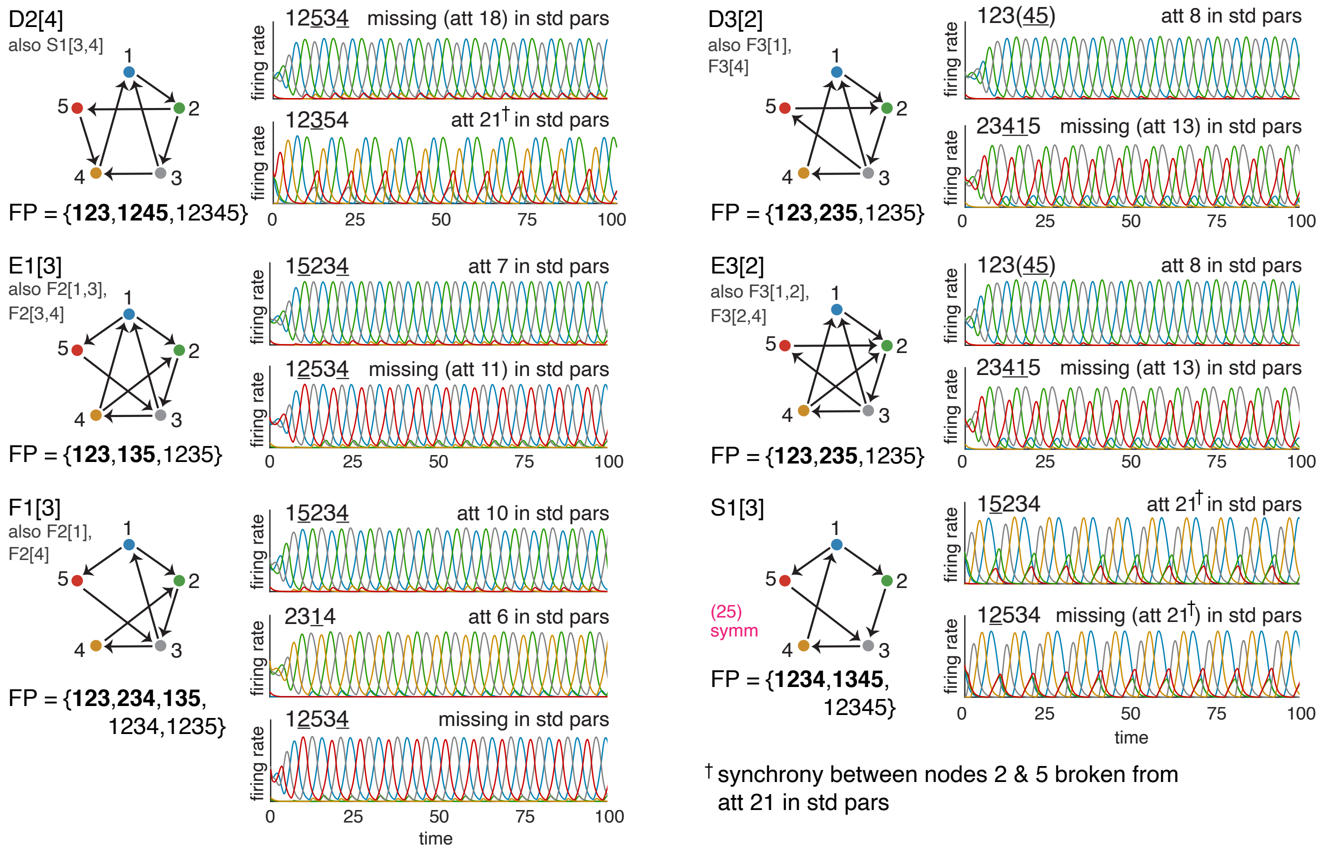

† synchrony between nodes 2 & 5 broken from att 21 in std pars

4. Classification of dynamic attractors for $\varepsilon = 0.25, \delta = 0.5$

Using the dictionary, we can now collect graphs that exhibit the same attractor into more specific graph families. It turns out that graphs with the same attractor fit nicely together into structural families defined by common and optional graph edges. Table 3 gives the sequence and the graph families corresponding to each of the 25 attractor classes. Note that "∼" indicates a forbidden edge. For example, D2[∼3,4,∗] represents the pair of graphs D2[1,4] and D2[4], which have no edge to node 3.

attractor	sequence	graph families	# graphs	# attractors	# ghosts
att 1	123	3-cycle + sources	30	30	0
att 2	1234	4-cycle + source (aka S0[∗])	5	5	0
att 3	12345	5-cycle	1	1	0
att 4	$123\underline{4}$	T4[∗]	7	8	0
att 5	$123\underline{4}$	D/E0[∗] & D/E4[∗]	30+14=44	30+14=44	0
att 6	$123\underline{4}$	F0[∗] & F4[∗]	11+7=18	22+7=29	0
att 7	$1\underline{5}23\underline{4}$	D1[2,∗], E1[∗], & D/E[1,4][2,∗]	4+7+4=15	4+7+4=15	0
att 8	$123(\underline{45})$	D/E3[∼4,∗]	5	5	0
att 9	$123\underline{54}$	D/E3[4,∗]	8	8	0
att 10	$1\underline{5}23\underline{4}$	F1[∗]	7	7	0
att 11	$12\underline{5}3\underline{4}$	F2[3,∗] & F[2,4][3]	3+1=4	4+1=5	1
att 12	$123\underline{54}$	F3[1,4,∗]	2	4	0
att 13	$123\underline{54}$	F3[∼1,4,∗]	2	0	2
att 14	$123(\underline{45})$	F3[2]	1	3	0
att 15	$12\underline{5}3\underline{4}$	F2[1,4]	1	4	0
att 16	$12\underline{5}3\underline{4}$	E2[1,4]	1	2	0
att 17	$12\underline{5}3\underline{4}$	E2[4]	1	1	0
att 18	$12\underline{5}3\underline{4}$	D2[∼3,4,∗]	2	2	1
att 19	$1\underline{5}23\underline{4}$	D1[4]	1	1	0
att 20	$1\underline{5}234$	S1[4]	1	1	0
att 21	1(25)34	S1[∼2,3,∗]	2	2	1
att 22	$1\underline{5}234$	S1[2,∗]	4	4	0
att 23	$1\underline{5}23\underline{5}4$	S[1,3][2,4]	1	1	0
att 24	$23(\underline{154})$	E[1,3][4,∗]	2	2	0
att 25	12534	E[1,2][3,4]	1	1	0

Table 3: Graph families for attractor classes of $n = 5$ oriented graphs with no sinks.

In the following pages, graph families for each attractor are depicted graphically, with dashed lines indicating optional edges. Fig 4 gives several examples of these *master graphs*, and the graph families they represent. In Fig 4A, all edges from the source node 5 back to the F graph are optional. Keeping in mind that 5 must have at least one outgoing edge, and the F graph has a (1,4) symmetry, we count 11 non-isomorphic graphs. In Fig 4A, all $2^3 - 1 = 7$ combinations of optional edges produce distinct graphs. The master graph in Fig 4C is the union of D3[∼4,∗] and E3[∼4,∗]: whether a graph has a D or E base is determined by the presence or absence of the $4 \to 2$ edge. Since 5 has two optional outgoing edges, there are 3 graphs with base D and 3 with base E in this family. However, the total number of non-isomorphic graphs is 5. This is because D3[1,2] ≅ E3[1], as shown in Fig 3. Finally, Fig 4D shows a master graph with only one optional edge. Since $5 \to 4$, both $5 \to 1$ and $5 \not\to 1$ options are allowed, yielding 2 graphs.

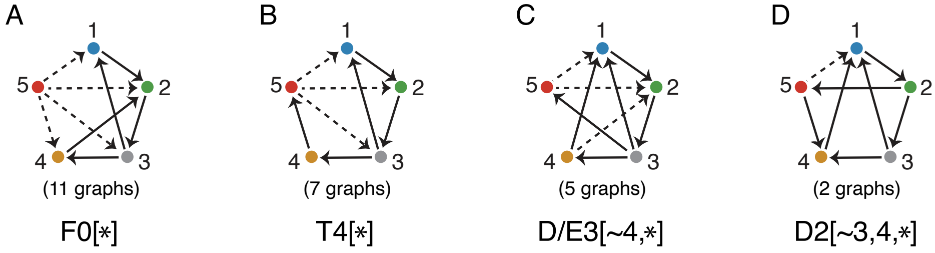

Figure 4: **Master graphs and their corresponding graph families.**

For each attractor class, in addition to showing the master graph(s) we also explicitly list all names for graphs that do not have sources, and were thus included in the dictionary. Moreover, we indicate with a superscript if the attractor in question was the primary or secondary attractor for the labeled graph (if a graph is listed without a superscript, it has a single core fixed point and a single attractor). For example, D2[1]^1 is listed under att 10, indicating that this attractor corresponds to the first core fixed point for D2[1], which is supported on 123 (see Dictionary). On the other hand, D2[1]^2 is listed under att 6, indicating that this attractor corresponds to the second core fixed point, supported on 125. Note that the att 6 and att 10 graph families are most naturally expressed with an F graph base, and D2[1] $\cong$ F1[2] $\cong$ F4[2].

It is important to keep in mind that the following classification of dynamic attractors was obtained for the standard parameters. At different parameters, some classes may split and others may merge. However, the prediction from core fixed points remains the same and $\mathrm{FP}(G)$ is invariant. Moreover, the graph families display structure that transcends the choice of parameters. It turns out that all ghost attractors also fit into one of these graph families, with the exception of F1[3]^3, corresponding to the third core fixed point supported on 135.

Attractor classes att 1–10

att 1 - pure 3-cycle (30 graphs, 30 attractors)

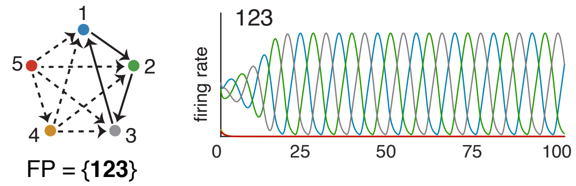

FP = {**123**}

att 2 - pure 4-cycle (S graph) (5 graphs, 5 attractors)

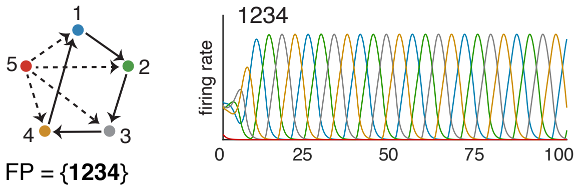

FP = {**1234**}

att 3 - pure 5-cycle (1 graph, 1 attractor)

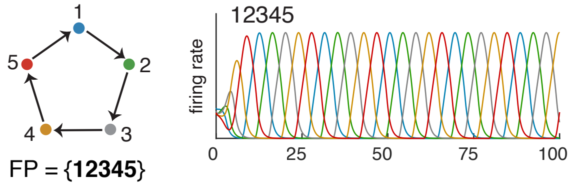

FP = {**12345**}

att 4 - pure tadpole (T graph) (7 graphs, 8 attractors)

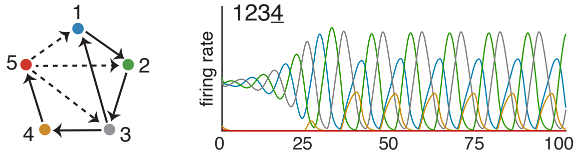

123 ∈ FP

T4[1], T4[2]^{1} (aka S1[4]^{2}), T4[3]^{1,2}, T4[1,2], T4[1,3], T4[2,3]^{1} (aka D1[4]^{2}), T4[1,2,3]

att 5 - pure D/E (44 graphs, 44 attractors)

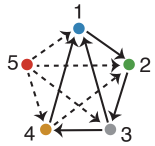

(30 graphs)

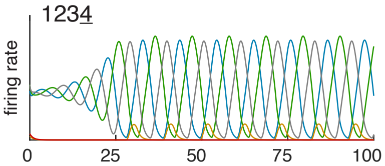

FP = {**123**}

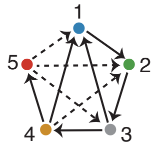

(14 graphs)

FP = {**123**}

D4[1], D4[2], D4[3], D4[1,2], D4[1,3], D4[2,3], D4[1,2,3], E4[1], E4[2], E4[3], E4[1,2], E4[1,3], E4[2,3], E4[1,2,3]

att 6 - pure F (18 graphs, 22+7=29 attractors)

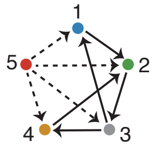

(11 graphs)

FP = {**123, 234**,1234}

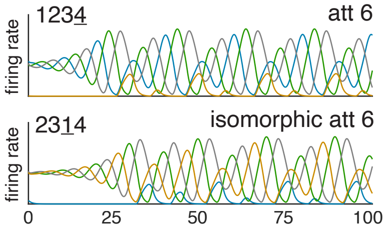

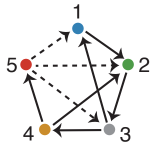

(7 graphs)

123, 234,1234 ∈ FP

F4[1]^{1}, F4[2]^{1}, F4[3]^{1}, F4[1,2]^{1}, F4[1,3]^{1}, F4[2,3]^{1}, F4[1,2,3]^{1}

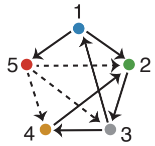

(7 isomorphic)

(isomorphic to F4[*] graphs)

123, 234,1234 ∈ FP

F1[2]^{2} (aka D2[1]^{2}), F1[3]^{2} (aka F2[1]^{2}), F1[4]^{2}, F1[2,3]^{2} (aka E2[1]^{2}), F1[2,4]^{2}, F1[3,4]^{2}, F1[2,3,4]^{2}

att 7 (15 graphs, 15 attractors)

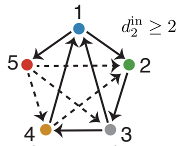

(11 graphs)

123 ∈ FP

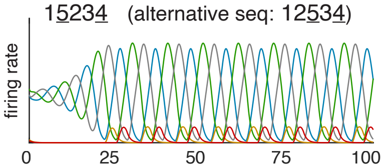

D1[2] (aka D2[3]), D1[2,3] (aka E2[3]), D1[2,4], D1[2,3,4], E1[2] (aka D2[1,3]), E1[3]^{1} (aka F2[1,3]^{1}, F2[3,4]^{2}), E1[4], E1[2,3] (aka E2[1,3]), E1[2,4], E1[3,4], E1[2,3,4]

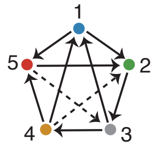

(4 graphs)

FP = {**123**}

D[1,4][2] (aka D2[3,4]), D[1,4][2,3] (aka E2[3,4]), E[1,4][2] (aka D2[1,3,4]), E[1,4][2,3] (aka E2[1,3,4])

att 8 (5 graphs, 5 attractors)

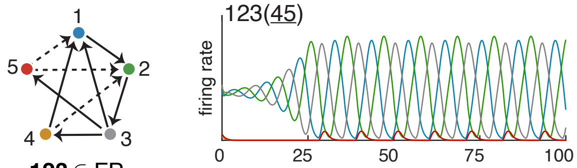

123 ∈ FP

D3[1], D3[2]^{1} (aka F3[1]^{1}, F3[4]^{2}), D3[1,2] (aka E3[1]), E3[1,2], E3[2]^{1} (aka F3[1,2]^{1}, F3[2,4]^{2})

att 9 (8 graphs, 8 attractors)

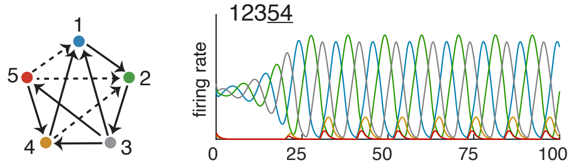

FP = {**123**}

D3[4], D3[1,4], D3[2,4], D3[1,2,4], E3[4], E3[1,4], E3[2,4], E3[1,2,4]

att 10 (7 graphs, 7 attractors)

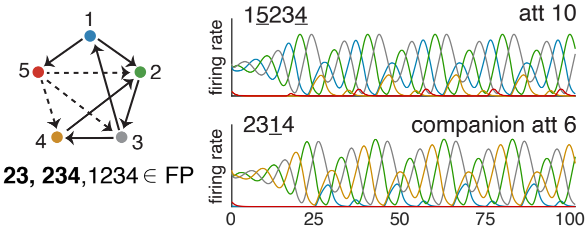

123, 234,1234 ∈ FP

F1[2]^{1} (aka D2[1]^{1}), F1[3]^{1} (aka F2[1]^{1}), F1[4]^{1}, F1[2,3]^{1} (aka E2[1]^{1}), F1[2,4]^{1}, F1[3,4]^{1}, F1[2,3,4]^{1}

Attractor classes att 11–19

att 11 (4 graphs, 5 attractors, 1 ghost attractor)

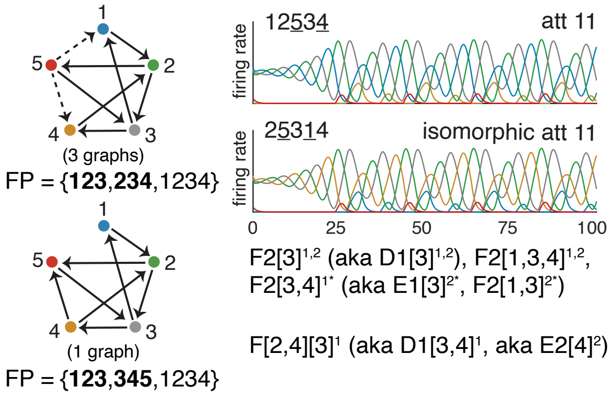

att 12 (2 graphs, 4 attractors)

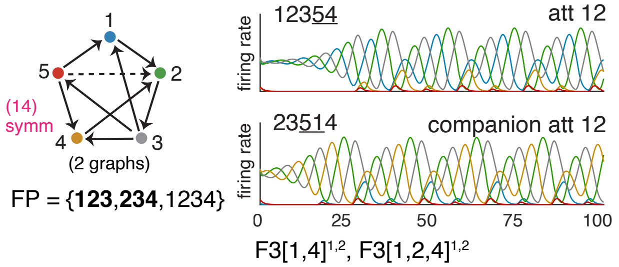

att 13 (2 graphs, 0 attractors, 2 ghost attractors)

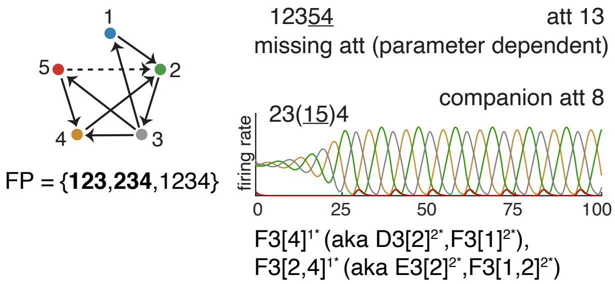

att 14 (1 graph, 3 attractors)

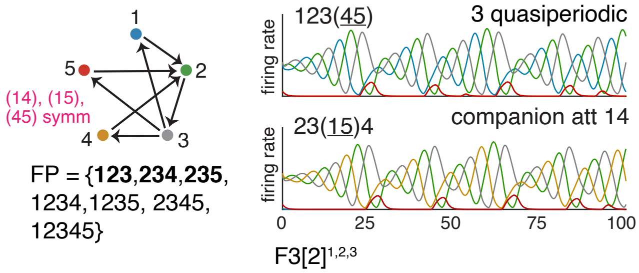

att 15 (1 graph, 4 attractors)

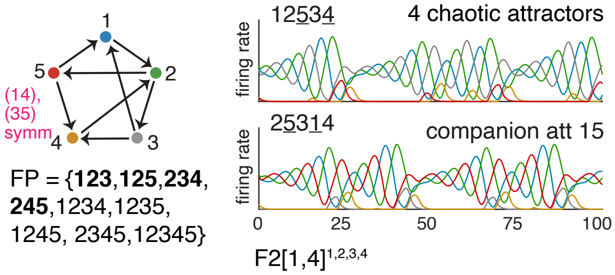

att 16 (1 graph, 2 attractors)

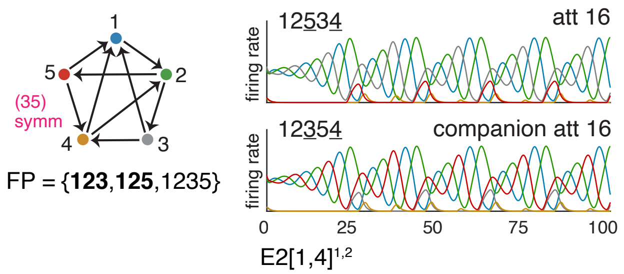

att 17 (1 graph, 1 attractor)

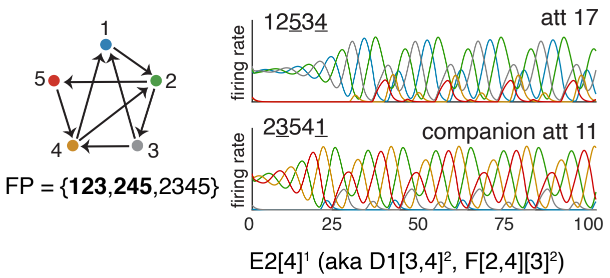

att 18 (2 graphs, 2 attractors, 1 ghost attractor)

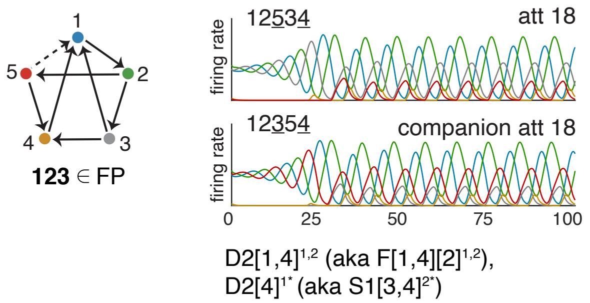

att 19 (1 graph, 1 attractor)

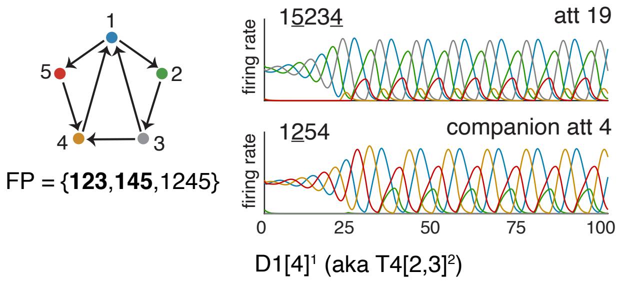

Attractor classes att 20–25

att 20 (1 graph, 1 attractor)

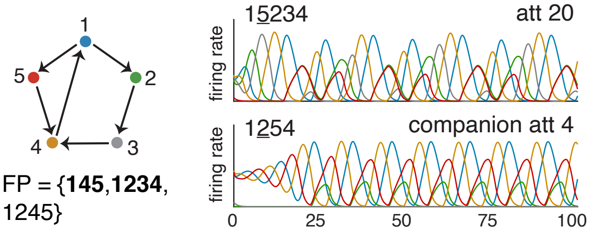

FP = {**145**,**1234**, 1245}

$S1[4]^{1}$ (aka $T4[2]^{2}$)

att 21† (2 graphs, 2 attractors, 1 ghost attractor)

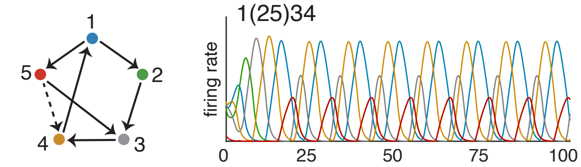

1234 ∈ FP

$S1[3]^{1 \vee 2^*}$, $S1[3,4]^{1}$ (aka $D2[4]^{2}$)

† synchrony between nodes 2&5 due to fusion of attractor for 1234 core with that for another core that involves nodes 1&5

att 22 (4 graphs, 4 attractors)

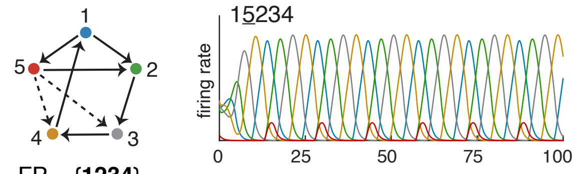

FP = {**1234**}

S1[2], S1[2,3], S1[2,4], S1[2,3,4]

att 23 (1 graph, 1 attractor)

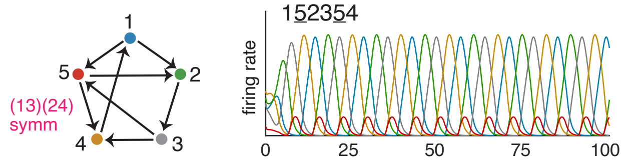

FP = {**12345**}

S[1,3][2,4]

att 24 (2 graphs, 2 attractors)

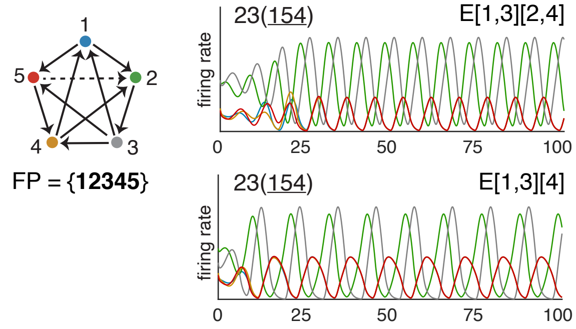

FP = {**12345**}

E[1,3][2,4], E[1,3][4]

att 25 (1 graph, 1 attractor)

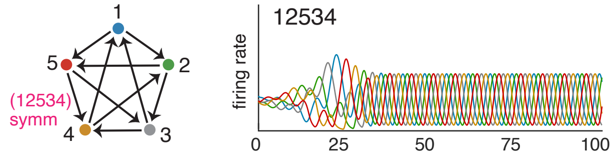

FP = {**12345**}

E[1,2][3,4]

redrawn as 5-star

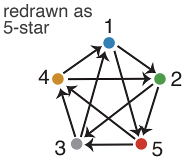

Ghost attractors:

$D2[4]^{1^*}$ (aka $S1[3,4]^{2^*}$),
$F3[4]^{1^*}$ (aka $D3[2]^{2^*}$, $F3[1]^{2^*}$),
$F3[2,4]^{1^*}$ (aka $E3[2]^{2^*}$, $F3[1,2]^{2^*}$),
$F2[3,4]^{1^*}$ (aka $E1[3]^{2^*}$, $F2[1,3]^{2^*}$),
$F2[4]^{1^*}$ (aka $F1[3]^{3^*}$, $F2[1]^{2^*}$),
$S1[3]^{2^*}$

References

[1] K. Morrison, A. Degeratu, V. Itskov, and C. Curto. Diversity of emergent dynamics in competitive threshold-linear networks: a preliminary report. Available at `https://arxiv.org/abs/1605.04463`

[2] C. Curto, J. Geneson, and K. Morrison. Fixed points of competitive threshold-linear networks. *Neural Comput.*, 31(1):94–155, 2019.
