# Supplementary material for: Core motifs predict dynamic attractors in combinatorial threshold-linear networks
Source: PLoS One. 2022 Mar 4;17(3):e0264456. doi: 10.1371/journal.pone.0264456 (PMC8896682; doi:10.1371/journal.pone.0264456)
Supplement: Attachment — Submitted filename: Parmelee_Moore_Morrison_Curto.pdf [file pone.0264456.s018.pdf]

Review of manuscript PONE-D-21-30804 ‘Core motifs predict dynamic attractors in combinatorial threshold-linear networks’ Caitlyn Parmelee, Samantha Moore, Katherine Morrison and Carina Curto

This paper explores the apparent correspondence between certain unstable fixed points of combinatorial threshold-linear networks (CTLNs) and stable dynamic attractors where the high-firing neurons in the attractor match the support of the fixed point in the underlying graph G. Guided by various examples, the authors hypothesise that the (static and dynamic) attractors of a CTLN with graph G correspond to the (stable and unstable) *core fixed points* of G. They also show that there are only certain subgraphs G_σ which can support core fixed points and they call these *core motifs*.

The aim is to demonstrate that core fixed points can predict a networks static and dynamic attractors, with stable core fixed points giving static attractors and unstable core fixed points having a corresponding dynamic attractor. In order to focus on dynamic attractors the authors consider only oriented graphs with no sinks which are known to have no stable fixed points. They determine all such graphs for $n = 5$, finding the core fixed points for each graph before carrying out extensive numerical simulations of the dynamics choosing initial conditions near each of the fixed points (core and others). They report that for one of the chosen parameter regimes the prediction of attractors from core fixed points was perfect, while for another ‘standard’ parameter set only 6 of the predicted 185 attractors were not realised (ghost attractors) and no spurious attractors were found for either parameter set. However, they did observe that networks with no core fixed points can still have dynamic attractors and that symmetric networks can have core fixed points that give rise to multiple attractors.

This work provides strong evidence that core motifs and core fixed points of the underlying graph can be used to predict the attractors of a CTLN and are therefore useful to connect network structure to dynamics. However, one has to keep in mind that CTLNs are only one class of network with fairly simple dynamics. For networks with more complicated node dynamics it is unlikely that such a direct correspondence between structure and observed attractors could be established. The interplay between the two is likely to be much more complex and subtle. That said, the work presented here is an interesting and worthy addition to the growing literature on the dynamics of CTLNs and may also be of interest to those

studying links between network structures and dynamics.

The paper is well-written, flows nicely and has well chosen examples to illustrate the concepts. However the paper is not entirely self-contained since there are numerous references to previous or concurrent work in references [9-12] which are used to determine the (core) fixed points. Although many of these 'graph rules' are described, some (in particular Lemma 21 of [11] and graphical domination - page 5 line 138) remain mysterious or left to the reader to find in reference [11]. It might be helpful to just include a brief summary of these two particular results. Many of the calculations are detailed in the extensive supplementary material. I regret that due to the number of calculations and length of the document I have not been able to verify these computations, but I have no reason to doubt their validity given the extensive knowledge the authors have on the dynamics of CTLNs.

Overall I would recommend this paper for publication without the need for any changes. However the authors may wish to consider the points below:

1. Page 3 line 71 The notation $[n]$ is first used but not defined until line 76.
2. Page 3 line 71 It would be helpful to point out that $x^* \in \mathbb{R}^n$. On line 72 x and x_i in the definition of the support should both have stars.
3. Page 7 line 174 It would be helpful to include a little reminder of the definition of a clique.
