# Supplementary material for: Core motifs predict dynamic attractors in combinatorial threshold-linear networks
Source: PLoS One. 2022 Mar 4;17(3):e0264456. doi: 10.1371/journal.pone.0264456 (PMC8896682; doi:10.1371/journal.pone.0264456)
Supplement: Attachment — Submitted filename: Response to Reviewers.pdf [file pone.0264456.s019.pdf]

Response to Reviewers

We would like to thank the reviewers for their careful reading of the manuscript and for their helpful comments, which we address below.

Reviewer 1 comments:

Reviewer #1: The authors of the paper study attractors in combinatorial Threshold-linear networks (CTLNs). Specifically, they investigate to what degree dynamic attractors in such systems correspond to so-called core fixed points.

CTLNs are a particular type of piecewise smooth neural network. It can be shown that (under some non-degeneracy conditions) fixed points can be labelled by subsets of the set of nodes. Core fixed points are then defined by certain minimality conditions on these subsets. A fixed point corresponds to an attractor if initial conditions close to the fixed point lead to the attractor in forward time, and if the dynamical behavior on the attractor involves strong activity exactly in the nodes of the subset corresponding to the fixed point. The hypothesis under investigation is that the core fixed points are precisely those that correspond to dynamic attractors.

This hypothesis is verified numerically on the set of all 5-node networks without sinks and without bi-directed links. This set is chosen because is it reasonably large (around 150 networks, up to isomorphic ones) and because they have only dynamic (as opposed to static) attractors. The results are in excellent agreement with the hypothesis, suggesting something intriguing is indeed going on. In addition, the attractors found can be classified in a relatively small number of cases, that furthermore agree to a very large extend with how the different networks can be built from certain set motifs.

It is my understanding that this paper represents a significant discovery concerning the extremely challenging search for the relation between interaction structure and dynamical behavior. It is furthermore written very well and can be read without much knowledge of the topic.

Some things to take into consideration are that I am not an expert in threshold linear networks, and am therefore not in a position to make a good judgement of the significance within this area. Moreover, (almost) all new results are numerical, and counter-examples of various sorts to the hypothesis are found. These are rare exceptions though, and furthermore analyzed in great detail. For instance, a different parameter regime is enough to locate missing attractors, and sometimes symmetry seems to create anomalous results (as is often the case in dynamical systems). It depends on journal policy if articles with solely numerical results are accepted. Rigorous results pertaining to the hypothesis might furthermore be very hard to prove (as is notorious for relations between network structure and dynamics, and considering the outliers found). Finally, there is always a risk that the observations found pertain only to networks with relatively small numbers of nodes. An example with a significantly larger number of nodes is presented though, where the hypothesis seems to hold.

In general, the results presented are very interesting. They are promising, not only in the field of TLNs, but also regarding the much wider field of network dynamical systems.

Some minor things:

Main document:

p. 2, l. 49: I realize terminology differs from field to field, but should "linear" not be "affine" here? This applies to more places.

We believe “linear” is correct here. Usually “linear systems of ODEs” refers to both homogeneous and non-homogeneous systems (where the right-hand side is an affine transformation).

p. 2, Figure 1: The sketch of [.]_+ in panel B is very minimalistic. Some information on the axis might make this a lot clearer.
We have added an explicit formula for the nonlinearity in the figure legend for panel (B) to clarify any confusion.

p.3, l. 76: [n] is already used on line 71, and so should probably be defined already there instead.
Good catch. The definition of [n] has now been moved to the first appearance.

p.3, l. 79: "is also fixed point" --> "is also a fixed point"
Thank you. Fixed.

p.3, l. 79: It becomes clear from context, but nevertheless it might be a good idea to shortly define subnetwork. I.e. they can have both incoming and outgoing arrows in the definition of the paper, it seems.
Thank you. A brief definition of subnetwork has now been added:

“A {\it subnetwork} supported on σ is a CTLN for the {\it induced subgraph} $G\vert_\sigma$ obtained from G by restricting to the vertices of σ and keeping only edges $i \to j$ for $i,j \in \sigma$”

p.4, l. 97: "corresponding to single" --> "corresponding to a single"
Thank you. Fixed.

p.5, l. 151: How could a fixed point that does not survive an embedding have a corresponding attractor?
This can happen with unstable fixed points. The corresponding attractor may be a periodic solution that is preserved as a trajectory in the larger state space even if the original fixed point of the subnetwork is not a fixed point in the larger network. In fact, we have seen examples of this behavior, though they are rare.

p.6, Figure 4: the last picture in panel B has a stray "fp" (whereas the others do not).
The “fp” stands for “fixed point,” and is intentionally included to emphasize that the initial conditions for that solution were initialized near the 1245 fixed point. We have now clarified the shorthand in the figure legend.

p.6, Figure 4: The reader learns only later why it says "263", instead of "236". A small mention that this is intentional and will be explained later might already clear it up.
Thank you for alerting us to the fact that this may be confusing. The first instance actually occurs in panel A, where we label the attractor for fixed point “235” as “high-firing 253.” We have now added the following sentence to the legend for panel A:

“The first attractor follows the cycle 125 in the graph, while the second one follows the cycle 253.”

p.11, l. 281: "oriented graph on n<=5, the set..." --> "oriented graph on n<=5 nodes, the set..."
Thank you. We have added in the word “nodes”.

p.11, l. 293: "and where they are localized within the network is the same" what is meant here? certainly exact numerical details will differ when parameters are changed?

We mean where they are localized combinatorially: the subset of high-firing neurons in the attractor is the same (though the numerical details will indeed differ). We have now changed the phrasing to say: “localized within the graph of the network.”

p.12, l. 358: "F attractor" --> "F attractors" (?)

We do refer to the “F attractor” in the singular.

Supp. Mat.:

p. 1: "We have verified that, with the exception of the 5-cycle, all of these graphs can be constructed by adding a single vertex to one of the five base graphs D, E, F, T, S, or the 3-cycle, shown in Figure 1A." This sentence implies you can get a graph with 5 nodes by adding a single vertex to the 3-cycle.
Good catch. We have now revised the text to say, “...all of these graphs can be constructed by adding a single vertex to one of the five base graphs D, E, F, T, S, shown in Figure1A, or two vertices to the 3-cycle.”

p.17: "and the F graph as a (1,4) symmetry" --> "and the F graph has a (1,4) symmetry"

Thank you. This has been fixed to “has”.

Reviewer 2 comments: This paper explores the apparent correspondence between certain unstable fixed points of combinatorial threshold-linear networks (CTLNs) and stable dynamic attractors where the high-firing neurons in the attractor match the support of the fixed point in the underlying graph G. Guided by various examples, the authors hypothesise that the (static and dynamic) attractors of a CTLN with graph G correspond to the (stable and unstable) core fixed points of G. They also show that there are only certain subgraphs G_σ which can support core fixed points and they call these core motifs.

The aim is to demonstrate that core fixed points can predict a networks static and dynamic attractors, with stable core fixed points giving static attractors and unstable core fixed points having a corresponding dynamic attractor. In order to focus on dynamic attractors the authors consider only oriented graphs with no sinks which are known to have no stable fixed points. They determine all such graphs for n = 5, finding the core fixed points for each graph before carrying out extensive numerical simulations of the dynamics choosing initial conditions near each of the fixed points (core and others). They report that for one of the chosen parameter regimes the prediction of attractors from core fixed points was perfect, while for another 'standard' parameter set only 6 of the predicted 185 attractors were not realised (ghost attractors) and no spurious attractors were found for either parameter set. However, they did observe that networks with no core fixed points can still have dynamic attractors and that symmetric networks can have core fixed points that give rise to multiple attractors.

This work provides strong evidence that core motifs and core fixed points of the underlying graph can be used to predict the attractors of a CTLN and are therefore useful to connect network structure to dynamics. However, one has to keep in mind that CTLNs are only one class of network with fairly simple dynamics. For networks with more complicated node dynamics it is unlikely that such a direct correspondence between structure and observed attractors could be established. The interplay between the two is likely to be much more complex and subtle. That said, the work presented here is an interesting and worthy addition to the growing literature on the dynamics of CTLNs and may also be of interest to those studying links between network structures and dynamics.

The paper is well-written, flows nicely and has well chosen examples to illustrate the concepts. However the paper is not entirely self-contained since there are numerous references to previous or concurrent work in references [9-12] which are used to determine the (core) fixed points. Although many of these 'graph rules' are described, some (in particular Lemma 21 of [11] and graphical domination - page 5 line 138) remain mysterious or left to the reader to find in reference [11]. It might be helpful to just include a brief summary of these two particular results. Many of the calculations are detailed in the extensive supplementary material. I regret that due to the number of calculations and length of the document I have not been able to verify these computations, but I have no reason to doubt their validity given the extensive knowledge the authors have on the dynamics of CTLNs.

Overall I would recommend this paper for publication without the need for any changes. However the authors may wish to consider the points below:

Although many of these `graph rules' are described, some (in particular Lemma

21 of [11] and graphical domination - page 5 line 138) remain mysterious or left to the reader to find in reference [11]. It might be helpful to just include a brief summary of these two particular results.

We seriously considered this, but decided it would be too technical and take up too much space in the paper for something that is only used/mentioned once. The reality is that we have many more graph rules than what we have listed in Table 1, but here we have selected only the essential ones that are broadly useful for our family of n=5 oriented graphs. This allows us to give the flavor of the graph rule-type analyses, while not overburdening the reader with too many technical details.

Graphical domination takes some time to explain, and we have devoted considerable space to it in reference [11] (also [10]). Lemma 21 is about the fixed point survival rules for the F graph (also called the "butterfly" graph), and it is similarly involved. We believe it will not be useful for the reader to see these explanations without going deeper into reference [11]. For the reviewers' benefit, we include some relevant excerpts from ref. [11] here.

Definition 5. *We say that* k *graphically dominates* j *with respect to* σ *if* $\sigma \cap \{j,k\} \neq \emptyset$ *and the following three conditions all hold:*

Fixed Points of Competitive Threshold-Linear Networks 109

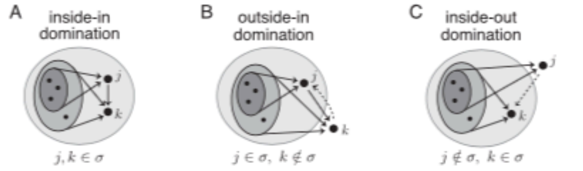

Figure 4: The three cases of graphical domination in Theorem 4. In each panel, k graphically dominates j with respect to σ (the outermost shaded region). The inner shaded regions illustrate the subsets of nodes that send edges to j and k. Note that the vertices sending edges to j are a subset of those sending edges to k, but this containment need not be strict. Dashed arrows indicate optional edges between j and k.

1. *For each* $i \in \sigma \setminus \{j,k\}$, *if* $i \to j$ *then* $i \to k$.
2. *If* $j \in \sigma$, *then* $j \to k$.
3. *If* $k \in \sigma$, *then* $k \not\to j$.

Theorem 4 *(graphical domination). Suppose* k *graphically dominates* j *with respect to* σ *(see Figure 4). Then the following statements all hold:*

1. *If* $j,k \in \sigma$, *then* $\sigma \notin FP(G|_\sigma)$, *and so* $\sigma \notin FP(G)$. *(inside-in domination)*
2. *If* $j \in \sigma$ *and* $k \notin \sigma$, *then* $\sigma \notin FP(G|_{\sigma\cup\{k\}})$, *and so* $\sigma \notin FP(G)$. *(outside-in domination)*
3. *If* $j \notin \sigma$, $k \in \sigma$, *and* $\sigma \in FP(G|_\sigma)$, *then* $\sigma \in FP(G|_{\sigma\cup\{j\}})$. *(inside-out domination)*

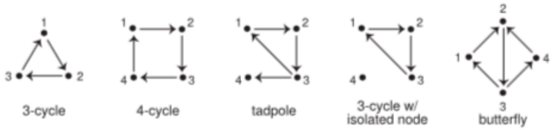

Figure 21: Permitted motifs of size $|\sigma| \leq 4$ (excluding independent sets) that can arise as subgraphs of an oriented graph with no sinks.

independent set can support a fixed point only if it is a union of sinks, and so no independent set will ever survive in a graph with no sinks.

The first three permitted motifs in Figure 21 are all uniform in-degree 1. Thus, the survival rule for these motifs is given by Rule 2: $\sigma \in FP(G)$ if and only if no external node $k \notin \sigma$ receives two or more edges from the nodes in σ.

The fourth motif is the disjoint union of a 3-cycle with an isolated node. Recall that a necessary condition for a disjoint union to survive is that each of its components survives (see Proposition 2). Since the isolated node cannot survive in a graph with no sinks, we see that this motif will never survive in our family of $n = 5$ graphs.

Finally, the butterfly graph is permitted by a parity argument. Since the graph has no sinks and no bidirectional edges, there are no fixed point supports of size less than or equal to 2. The only permitted motifs of size 3 are the two 3-cycles—123 and 234—which both survive by Rule 2. Thus, by parity, the full graph must be a fixed point support. The survival rules for the butterfly graph are summarized in the following lemma.

Lemma 21. *Let* $\sigma = \{1,2,3,4\}$, *and let G be any graph such that* $G|_\sigma$ *is the butterfly graph in Figure 21. Then* $\sigma \in FP(G)$ *if and only if every* $k \notin \sigma$ *either receives at most one edge from* σ, *or receives two edges from among nodes 1, 2, and 4.*

Proof. To derive the butterfly graph's survival rules, let $\sigma = \{1,2,3,4\}$, and consider all possible configurations of outgoing edges from the σ to an added node 5. It is easy to check that if node 5 receives fewer than two edges from σ, there will always be inside-out graphical domination, and so σ will survive. In contrast, if node 5 receives three or more edges from σ, then 5 will outside-in dominate some node in σ, and so σ will not survive.

When node 5 receives exactly two edges from σ, survival is more complicated and depends on which nodes 5 receives from. There are four cases: (i) $1,3 \to 5$ (equivalently $3,4 \to 5$ by symmetry): 5 outside-in dominates 1, and so σ does not survive; (ii) $2,3 \to 5$: 5 outside-in dominates 3, and so

1. Page 3 line 71 The notation [n] is first used but not defined until line 76.
Thank you. The definition of [n] was moved to this first appearance.

2. Page 3 line 71 It would be helpful to point out that x* is in R_n. On line 72 x and x_i in the definition of the support should both have stars.
Thank you. We have made these changes.

3. Page 7 line 174 It would be helpful to include a little reminder of the definition of a clique.
Agreed. We have added "(all-to-all bidirectionally connected subgraphs)" after the word "cliques".

Requests from PLOS One Editors:

Please ensure that your manuscript meets PLOS ONE's style requirements, including those for file naming.

Please ensure that you include a title page within your main document.

Done.

We note that you have provided additional information within the Acknowledgements Section that is not currently declared in your Funding Statement. Please note that funding information should not appear in the Acknowledgments section or other areas of your manuscript. We will only publish funding information present in the Funding Statement section of the online submission form.

The statement of funding sources was removed from the Acknowledgements section. Here is the updated Funding Statement that the journal should use:

"This work was supported by NIH R01 EB022862 (CC & KM), NIH R01 NS120581 (CC), NSF DMS-1951165 (CC), and NSF DMS-1951599 (KM). The funders had no role in study design, data collection and analysis, decision to publish, or preparation of the manuscript."

Upon re-submitting your revised manuscript, please upload your study's minimal underlying data set as either Supporting Information files or to a stable, public repository and include the relevant URLs, DOIs, or accession numbers within your revised cover letter.

Please review your reference list to ensure that it is complete and correct.
